# Potassium channels Kv1.3 and KCa3.1 cooperatively and compensatorily regulate antigen-specific memory T cell functions

**DOI:** 10.1038/ncomms14644

**Published:** 2017-03-01

**Authors:** Eugene Y. Chiang, Tianbo Li, Surinder Jeet, Ivan Peng, Juan Zhang, Wyne P. Lee, Jason DeVoss, Patrick Caplazi, Jun Chen, Søren Warming, David H. Hackos, Susmith Mukund, Christopher M. Koth, Jane L. Grogan

**Affiliations:** 1 Department of Immunology, Genentech Inc., 1 DNA Way, South San Francisco, California 94080, USA; 2 Department of Biochemical and Cellular Pharmacology, Genentech Inc., 1 DNA Way, South San Francisco, California 94080, USA; 3 Department of Translational Immunology, Genentech Inc., 1 DNA Way, South San Francisco, California 94080, USA; 4 Department of Pathology, Genentech Inc., 1 DNA Way, South San Francisco, California 94080, USA; 5 Department of Molecular Biology, Genentech Inc., 1 DNA Way, South San Francisco, California 94080, USA; 6 Department of Neurobiology, Genentech Inc., 1 DNA Way, South San Francisco, California 94080, USA; 7 Department of Structural Biology, Genentech Inc., 1 DNA Way, South San Francisco, California 94080, USA

## Abstract

Voltage-gated Kv1.3 and Ca^2+^-dependent KCa3.1 are the most prevalent K^+^ channels expressed by human and rat T cells. Despite the preferential upregulation of Kv1.3 over KCa3.1 on autoantigen-experienced effector memory T cells, whether Kv1.3 is required for their induction and function is unclear. Here we show, using Kv1.3-deficient rats, that Kv1.3 is involved in the development of chronically activated antigen-specific T cells. Several immune responses are normal in Kv1.3 knockout (KO) rats, suggesting that KCa3.1 can compensate for the absence of Kv1.3 under these specific settings. However, experiments with Kv1.3 KO rats and Kv1.3 siRNA knockdown or channel-specific inhibition of human T cells show that maximal T-cell responses against autoantigen or repeated tetanus toxoid stimulations require both Kv1.3 and KCa3.1. Finally, our data also suggest that T-cell dependency on Kv1.3 or KCa3.1 might be irreversibly modulated by antigen exposure.

## Abstract (web summary)

Potassium channels are essential for modulating T-cell functions. Here, by characterizing rat models and analysing human T cells, the authors identify differential requirements of two potassium channel proteins, Kv1.3 and KCa3.1, for the induction of conventional versus autoreactive T-cell responses.

A hallmark of the adaptive immune system is the generation of long-lived, self-renewing memory T cells in response to pathogen-derived antigenic stimuli. Electrophysiology studies have implicated the potassium ion (K^+^) channel Kv1.3 as having a critical role in the regulation of chronically activated effector memory T (T_EM_) cell immune responses. K^+^ channels are tetrameric membrane proteins that selectively conduct K^+^ across cellular membranes. Out of the 80 distinct K^+^ channel genes that have been identified in the human genome, only two are dominantly expressed on human T cells; these are the homotetramers of the Shaker-related voltage-gated Kv1.3 (*Kcna3*) and the Ca^2+^-dependent KCa3.1 K^+^ channels (*Kcnn4*)^1^. These channels provide the K^+^ efflux necessary to counter-balance the Ca^2+^ influx crucial for T-cell activation^1^,^2^,^3^,^4^.

Functional expression of Kv1.3 and KCa3.1 on T-cell subsets has been extensively characterized using single-cell patch-clamp technique under channel-specific conditions^1^,^5^,^6^,^7^,^8^,^9^. At rest, T cells express low levels of both K^+^ channels, and both are upregulated upon antigen-specific or mitogen-specific activation. However, chronically activated T_EM_ cells and autoreactive T cells have been reported to preferentially express Kv1.3. The differential expression of these ion channels results in different sensitivity of naive versus autoreactive memory T cells to selective blockers of Kv1.3 and KCa3.1, such as peptide toxin ShK and its derivatives^10^,^11^,^12^,^13^,^14^,^15^,^16^ and pharmacological inhibitor TRAM-34 (refs 17, 18), respectively.

As single-cell patch clamp revealed that autoreactive T_EM_ cells predominantly utilize Kv1.3, Kv1.3 inhibitors have been evaluated for suppression of autoimmune reactions. Inhibition of Kv1.3 with ShK or its derivatives has shown partial efficacy in preclinical autoimmune disease rat models including experimental autoimmune encephalomyelitis^15^,^19^,^20^, pristane-induced arthritis^15^, and experimental autoimmune diabetes^15^, as well as in T-cell-dependent models of delayed-type hypersensitivity (DTH)^15^,^21^, allergic contact dermatitis^22^,^23^ and asthma^24^. Despite the ability of Kv1.3 inhibitors to impede preclinical models of autoimmune disease, it is not clear whether inhibition of Kv1.3 is sufficient to fully inhibit pathological T-cell activation and effector function. In the context of single cells, channel blockers abrogate Kv1.3 channel function in patch-clamp experiments. However, only partial inhibition of *in vitro* T-cell responses has been detected using functional readouts, such as proliferation or cytokine production^12^,^13^,^14^,^15^,^16^,^20^,^25^,^26^. This observation raises the possibility that ion channels other than Kv1.3, such as KCa3.1, may have functional activity.

Mouse T cells, unlike rat or human T cells, co-express additional Kv1 channel family members, including Kv1.1, Kv1.4 and Kv1.6 (ref. 27), rendering Kv1.3 redundant, and thereby precludes the translation of mouse T-cell function to humans^7^. Conversely, selective K^+^ channel expression in rat T cells phenocopies human T cells^7^. Thus, in order to characterize the role of Kv1.3 in T-cell responses, we generated a *Kcna3* knockout (KO) rat. Characterization of functional responses in *Kcna3*^*−/−*^ rats compared with wild-type (WT) rats, together with the use of channel-specific blockers and *in vitro* antigen recall assays, enables us to assess the individual contributions of Kv1.3 and KCa3.1, providing a more comprehensive analysis of the role of these K^+^ channels in T-cell function than enabled by electrophysiology methods. These approaches reveal that inhibition of Kv1.3 alone is insufficient to inhibit functional T-cell responses and, moreover, that KCa3.1 compensates for the loss of Kv1.3 in *Kcna3*^*−/−*^ rats. Our rat data are translatable to human T cells, as differential utilization of Kv1.3 or KCa3.1 is detected in pathogen-specific T cells as compared with autoreactive T cells, with skewing towards Kv1.3 dependency resulting from repeated antigen stimulation. Collectively, our study demonstrates that repeated exposure to specific antigen might affect whether Kv1.3 or KCa3.1 functionally predominates, and that Kv1.3 and KCa3.1 have complementary and compensatory roles, thereby providing redundant mechanisms to ensure T-cell activation.

## Results

### *KCNA3* ^*−*/*−*^ rat T cells are functionally competent

**Figure 1:**
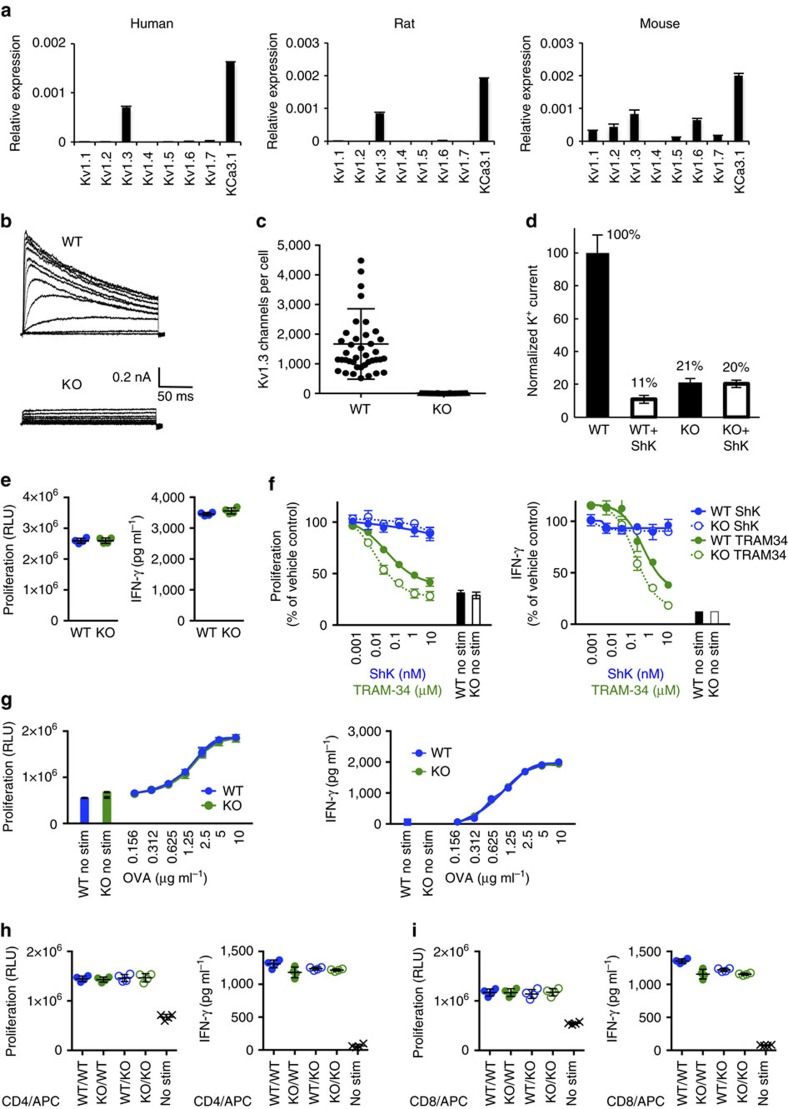
Characterization of *Kcna3*^*−*/*−*^ T cells. (**a**) K^+^-channel expression in human, rat and mouse T cells. Gene expression of Kv1 family members and KCa3.1 in naive CD4^+^ T cells from human (left), rat (centre) or mouse (right). Relative expression was determined by normalizing to housekeeping gene RPL19. Electrophysiological and pharmacological tests show null Kv1.3 channel in *Kcna3*^*−*/*−*^ T cells. (**b**) Representative voltage-currents from WT and *Kcna3*^*−*/*−*^ T cells. Currents were elicited by depolarizing voltage steps from −60 to +40 mV (10 mV increments every 30 s, with −80 mV membrane-holding potential). (**c**) Kv1.3 channel number in WT (*n*=40) and *Kcna3*^*−*/*−*^ (*n*=50) T cells after 48 h activation. The mean Kv1.3 channel numbers were 1667±187 in WT and undetectable in *Kcna3*^*−*/*−*^ T cells. (**d**) Normalized WT and *Kcna3*^*−*/*−*^ T cell K^+^ currents before and after Shk inhibition. 89% WT T cell K^+^ current was blocked by 1 nM Shk, but no Shk-sensitive current was detected in *Kcna3*^*−*/*−*^ T cells. *Kcna3*^*−*/*−*^ T-cell responses to activation. (**e**) Proliferation (left) and IFN-γ (right) responses to anti-CD3 and anti-CD28 stimulation. Spleen cells from WT or *Kcna3*^*−*/*−*^ rats were stimulated for 3 days. Individual biological replicates (*n*=4 per group) are shown with mean±s.d. (**f**) Effects of ShK and TRAM-34 on polyclonal T-cell activation. Data are shown as mean±s.d. (*n*=4 biological replicates per group). (**g**) OVA-specific T-cell proliferation responses. Draining lymph node and spleen cells from OVA-immunized WT and *Kcna3*^*−*/*−*^ rats were plated at a 1:10 lymph node:spleen cell ratio and stimulated *in vitro* with OVA at various concentrations. Data are shown as mean±s.d. (*n*=4 biological replicates per group). (**h**,**i**) *Kcna3*^*−*/*−*^ rat dendritic cell competency. CD4^+^ (**h**) or CD8^+^. (**i**) T cells isolated from DLN of OVA-immunized WT (blue) and *Kcna3*^*−*/*−*^ (green) rats were co-cultured with APCs from WT (filled circles) or *Kcna3*^*−*/*−*^ (open circles) rats and stimulated with OVA. Proliferation responses were determined at day 3 of culture. Individual biological replicates (*n*=4 per group) are shown with mean±s.d.

Mice are not suitable for exploring the role of Kv1.3 and KCa3.1 in T-cell responses due to the expression and redundancy of many Kv1 channel family members in the immune compartment^7^,^27^,^28^. Rats, however, are phenotypically similar to humans in terms of Kv1.3 being the only Kv1 member expressed by T cells (Fig. 1a). Therefore, we generated a rat deficient of Kv1.3 (*Kcna3*^*−*/*−*^) using zinc finger nuclease targeted deletion on Dark Agouti rats (Supplementary Fig. 1a–c) to ask whether Kv1.3 was required for functional T-cell responses. *Kcna3*^*−*/*−*^ rats appeared phenotypically normal and displayed no gross abnormalities. Analysis of K^+^ channel mRNA expression confirmed that the *Kcna3*^*−*/*−*^ rat CD4^+^ and CD8^+^ T cells did not express *Kcna3* transcripts, nor did they express other Kv1 family genes; only KCa3.1 transcripts (*Kcnn4*) were detectable (Supplementary Fig. 1d). Absence of Kv1.3 protein was validated by flow cytometry (Supplementary Fig. 1e). Electrophysiology provided functional confirmation that Kv1.3 was deleted, as Kv1.3-dependent currents were undetectable (Fig. 1b–d). Detailed characterization of the immune compartment revealed no differences in T- or B-cell populations in *Kcna3*^*−*/*−*^ versus WT rats (Supplementary Fig. 2). Polyclonal activation of splenic T cells with anti-CD3 and anti-CD28 *in vitro* revealed no differences between *Kcna3*^*−*/*−*^ and WT rat T cells using proliferation and effector cytokine production as functional readouts (Fig. 1e). Consistent with published findings for human T cells, the KCa3.1-specific small molecule inhibitor TRAM-34 inhibited naive WT rat T-cell proliferation in response to polyclonal activation, whereas this response was unaffected by the Kv1.3-specific small molecule inhibitor ShK; IFN-γ production was similarly inhibited by TRAM-34 but not ShK (Fig. 1f). As expected, ShK had no effect on *Kcna3*^*−*/*−*^ T-cell responses; inhibition with TRAM-34 was slightly enhanced in *Kcna3*^*−*/*−*^ as compared with WT.

To assess antigen-specific T-cell responses, *Kcna3*^*−*/*−*^ and WT rats were immunized with ovalbumin (OVA) and then *in vitro* antigen recall assays were performed. *Kcna3*^*−*/*−*^ T-cell recall responses to titred doses of OVA antigen were comparable to WT (Fig. 1g). Antigen presentation in *Kcna3*^*−*/*−*^ rats was also fully competent, as the ability of antigen-presenting cells (APC) derived from OVA-immunized *Kcna3*^*−*/*−*^ rats to mediate CD4^+^ and CD8^+^ T-cell OVA-specific recall responses was similar to WT, and vice versa (Fig. 1h,i). These data suggest that Kv1.3 is not required for the appropriate development of antigen-specific T-cell responses.

### *Kcna3* ^*−*/*−*^ rats mount normal immune responses *in vivo*

**Figure 2:**
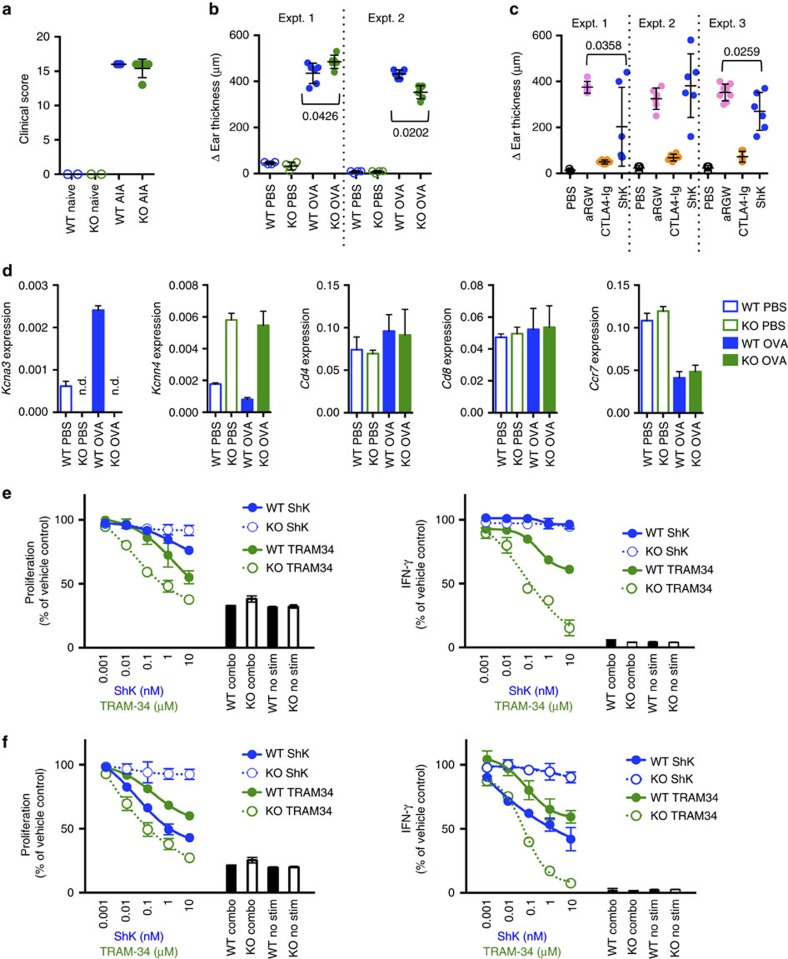
AIA and DTH response in *Kcna3*^*−*/*−*^ rats. (**a**) WT (blue) or *Kcna3*^*−*/*−*^ (green) rats were given a single injection of CFA to induce AIA (filled symbols, *n*=5 biological replicates per group) or were untreated (open symbols, naive, *n*=2 per group) and clinical score assessed at day 21. Individual animals are represented by discrete symbols and mean±s.d. are shown. Delayed-type hypersensitivity. (**b**) WT (blue) or *Kcna3*^*−*/*−*^ (green) OVA-immunized rats were subsequently challenged with OVA (filled symbols, *n*=6 biological replicates per group) or PBS (open symbols, *n*=4 per group) and ear swelling was measured 24 h later. Individual animals are represented by discrete symbols and mean±s.d. are shown. Experiment was performed twice, with each experiment delineated by the dotted line. Statistically significant differences are denoted with *P* values as determined by Student's *t-*test. (**c**) Effect of Kv1.3 blockade on DTH inflammatory responses. WT rats were immunized with OVA then subsequently challenged 1 week later with either PBS or OVA. OVA-rechallenged animals were treated with control anti-ragweed (aRGW) antibody (pink), CTLA4-Ig (orange) or ShK (blue). Ear swelling was measured 24 h later. Individual biological replicates (n=6 per group) are shown with mean±s.d. Experiment was performed three times, with each experiment delineated by dotted lines. Statistically significant differences between ShK and anti-ragweed control groups are denoted with *P* values; CTLA4-Ig inhibition was statistically significant in all experiments (*P*<0.0001). (**d**) Relative gene expression of *Kcna3* (Kv1.3), *Kcnn4* (KCa3.1), *Cd4*, *Cd8* or *Ccr7* was determined on bulk DLN cells by normalizing to housekeeping gene *Rpl19*. Data are shown as mean±s.d. (**e**,**f**) OVA-specific *in vitro* recall response from OVA-immunized rats with PBS (**e**) or OVA (**f**) secondary challenge. Proliferation (left) and IFN-γ (right) responses were determined in the absence or presence of ShK, TRAM-34 or a combination of both (‘combo'). ‘No stim' denotes unstimulated cell conditions (absence of OVA antigen). Data are shown as mean±s.d.

To determine the functional consequence of the loss of Kv1.3 on T-cell responses *in vivo*, we employed rat models of adjuvant-induced arthritis (AIA) and DTH. AIA is induced by a single injection of complete Freund adjuvant (CFA), and is a model of human rheumatoid arthritis with CD4^+^ T cells having an important role in disease initiation and maintenance^29^,^30^,^31^. *Kcna3*^*−*/*−*^ rats developed AIA in a manner similar to WT rats, indicating that there were no defects in T-cell activation (Fig. 2a). At day 21, both groups exhibited severe disease with clinical scores of 16.0±0.0 and 15.4±0.6 in WT and KO rats, respectively. DTH is an acute inflammatory immune response initiated by the activation of tissue-resident CD4^+^ T_EM_ cells following rechallenge with antigen^21^,^32^. *Kcna3*^*−*/*−*^ rats mounted an OVA-specific DTH response measurable in the ear that was comparable to WT rats (Fig. 2b). In this model, ShK treatment has been reported to reduce ear swelling when administered during the effector phase, suggesting that blockade of Kv1.3 impairs T-cell-mediated inflammation^15^. In contrast to published reports^15^, ShK did not inhibit DTH in our studies, consistent with our observations in *Kcna3*^*−*/*−*^ rats (Fig. 2c).

Analysis of *Kcnn4* expression by mRNA showed that KCa3.1 was significantly upregulated in T cells from *Kcna3*^*−*/*−*^ rats both after OVA immunization and OVA-challenge relative to WT rats (a three- to six-fold increase), whereas CD4 and CD8 mRNA levels were unaffected (Fig. 2d). Relative CCR7 expression was comparable between WT and KO, with decreased CCR7 in the OVA-rechallenged rats compared with those receiving PBS, indicating a larger T_EM_ cell fraction (Fig. 2d). Since the expectation was for *Kcna3*^*−*/*−*^ rats to be incapable of mounting an effector response, these results suggest that KCa3.1 can compensate for the absence of Kv1.3.

*In vitro* OVA-specific recall responses were performed to determine if *Kcna3*^*−*/*−*^ T-cell responses were dependent on KCa3.1. Draining LN and spleen cells from WT or *Kcna3*^*−*/*−*^ rats with DTH were stimulated *in vitro* with OVA. Antigen-specific recall responses from WT and *Kcna3*^*−*/*−*^ rats were comparable in both proliferation and IFN-γ production (Supplementary Fig. 3). As *Kcna3*^*−*/*−*^ T cells mounted fully competent responses, expression of KCa3.1 was sufficient for T-cell activation. To test whether *Kcna3*^*−*/*−*^ T cells were solely dependent on KCa3.1, OVA-specific recall responses were performed in the presence of ShK, TRAM-34 or a combination of both. Despite treating cells with ShK and TRAM-34 at concentrations well above their reported *K*_d_ values (ShK *K*_d_=10 pM (ref. 12), used at 10 nM; TRAM-34 *K*_d_=20 nM (ref. 18), used at 10 μM), full inhibition of proliferation or IFN-γ was not achieved. ShK did not affect OVA-specific recall responses from PBS-rechallenged WT rats (Fig. 2e), but did inhibit recall responses from OVA-rechallenged rats, albeit not completely (Fig. 2f). TRAM-34 alone partially inhibited proliferation and IFN-γ production by either PBS- or OVA-rechallenged T cells (Fig. 2e,f). The combination of ShK and TRAM-34 completely inhibited T-cell responses. For *Kcna3*^*−*/*−*^ T cells, TRAM-34 inhibitory effects were more robust and full inhibition was observed at the highest concentrations under both rechallenge conditions.

### Repeated antigen-specific stimulation skews to Kv1.3 dependency

**Figure 3:**
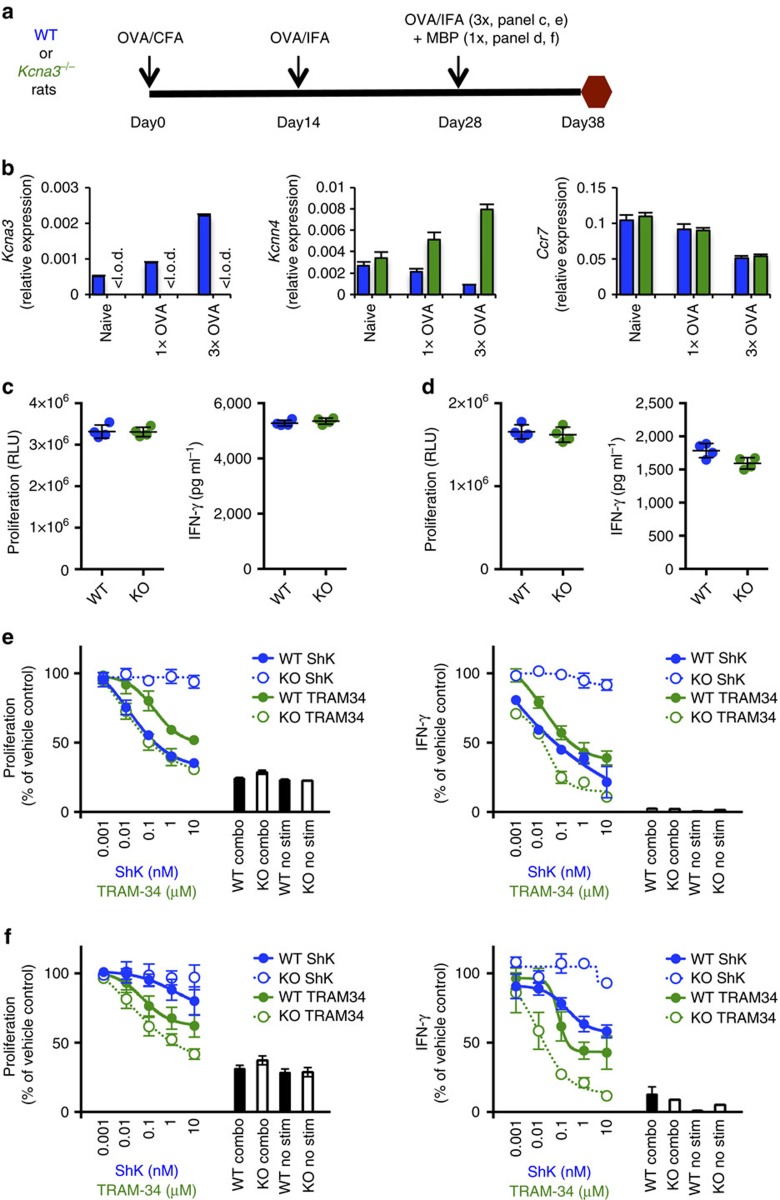
Antigen-specific effector T cells develop normally in K*cna3*^*−*/*−*^ rats. (**a**) Experimental design. WT or *Kcna3*^*−*/*−*^ rats (*n*=4 per group) were immunized three times with OVA and one time with MBP, administered concurrently during the final immunization. (**b**) Relative expression of *Kcna3* (Kv1.3), *Kcnn4* (KCa3.1) or *Ccr7* in CD4^+^ T cells isolated from DLN of WT (blue) or *Kcna3*^*−*/*−*^ (green) rats was calculated by normalizing to housekeeping gene *Rpl19*. Data are shown as mean±s.d. ‘<l.o.d.' denotes below limit of detection. (**c**,**d**) *In vitro* recall stimulation was performed using cells harvested from WT (blue) or *Kcna3*^*−*/*−*^ (green) rats immunized three times with OVA and one time with MBP, with OVA (**c**) or MBP (**d**) as antigen and proliferation (left) and IFN-γ (right) responses determined after 3-day stimulation. Individual biological replicates (*n*=4 per group) are shown with mean±s.d. (**e**,**f**) Effects of ShK (blue), TRAM-34 (green) and inhibitor combination on T-cell responses to *in vitro* restimulation with OVA (**e**) or MBP (**f**). For combination treatment (combo), cells were cultured with 10 nM ShK and 10 μM TRAM-34. ‘No stim' denotes unstimulated cell conditions. Data are shown as mean±s.d. (*n*=4 biological replicates per group).

Sensitivity of T cells to ShK increased in DTH rats that received secondary challenge with OVA but not rechallenged with PBS (Fig. 2f). To examine if repeated antigen-specific stimulation would bias T-cell K^+^ channel dependency from KCa3.1 towards Kv1.3, WT and *Kcna3*^*−*/*−*^ rats were immunized with two different antigens. One antigen, OVA, was administered three times, while the second antigen, myelin basic protein (MBP), was given once, together with OVA in the last immunization (Fig. 3a). In WT draining lymph node CD4^+^ T cells, *Kcna3* transcript levels increased approximately five-fold after three rounds of repeated antigen stimulation, compared with *Kcnn4* levels that decreased nearly 70% with repeated OVA stimulation (Fig. 3b). In contrast, expression of KCa3.1 in *Kcna3*^*−*/*−*^ CD4^+^ T cells increased with each immunization, doubling after three rounds. CCR7 expression in primary OVA-immunized rats was similar to expression in naive CD4^+^ T cells, but was reduced following the third round of immunization, and no difference was detected between WT versus *Kcna3*^*−*/*−*^ rats (Fig. 3b). Frequencies of CD4^+^ and CD8^+^ T cells in WT and *Kcna3*^*−*/*−*^ rat spleens or draining lymph nodes were similar after single or repeated OVA immunization (Supplementary Fig. 4).

To determine if the changes in expression observed at the transcriptional level were reflected in functional protein expression, we examined the sensitivity of T cells to specific inhibitors of Kv1.3 and KCa3.1. Upon *in vitro* stimulation, WT and *Kcna3*^*−*/*−*^ T-cell responses were similar against both OVA (Fig. 3c) and MBP (Fig. 3d). ShK inhibited T-cell responses from WT animals repeatedly immunized with OVA (Fig. 3e) but not from WT animals receiving single immunization with MBP (Fig. 3f). In contrast, TRAM-34 inhibited, albeit not completely, WT T-cell responses regardless of antigen and TRAM-34 effects were enhanced in *Kcna3*^*−*/*−*^ T cells. Complete abrogation of both OVA-specific and MBP-specific WT T-cell responses was observed only with combination treatment with both ShK and TRAM-34, a result achieved in *Kcna3*^*−*/*−*^ T cells with TRAM-34 alone (Fig. 3e,f).

### Human T cells gain Kv1.3 dependency with repeated stimulation

**Figure 4:**
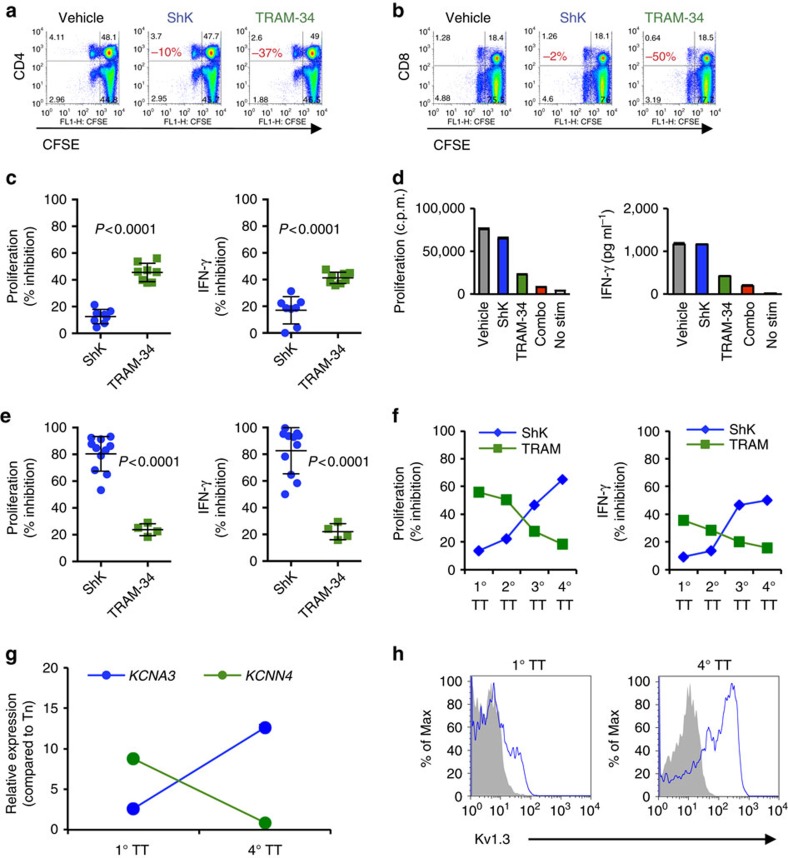
Full inhibition of antigen-specific human T cells requires blockade of both Kv1.3 and KCa3.1. (**a**,**b**) Inhibition of CD4^+^ (**a**) or CD8^+^ (**b**) T-cell proliferation response to TT stimulation as determined by CFSE dilution. One representative donor is shown. Percentages indicated in red denote % reduction in proliferation relative to vehicle-treated cells. Primary (1°) T-cell response to TT was determined in the absence or presence of either 10 nM ShK (*n*=8 biological replicates) or 1 μM TRAM-34 (*n*=9). (**c**) Proliferation responses were determined at day 4; IFN-γ concentrations were measured at day 3. % inhibition was determined by comparing proliferation or IFN-γ concentration in the presence of inhibitor to vehicle control. Data are shown as individual data points with mean±s.d. Statistically significant differences are denoted with *P* values as determined by Student's *t-*test. (**d**) Inhibition of both Kv1.3 and KCa3.1 fully abrogates TT-specific T cell responses. PBMC from T1D donors were stimulated with TT in the absence or presence of 10 nM ShK, 1 μM TRAM-34 or a combination of both (combo). ‘No stim' denotes absence of TT. Data are shown as mean±s.d. of replicate wells and are representative of three independent experiments. (**e**) Proliferation and IFN-γ production of TT-specific T cells following four rounds of stimulation (4°). After three rounds of TT stimulation, cells were rested and then restimulated in the presence of irradiated autologous PBMC with TT in the absence or presence of either 10 nM ShK (*n*=11 biological replicates) or 1 μM TRAM-34 (*n*=4). (**f**) Effect of ShK or TRAM-34 on proliferation or IFN-γ production of TT-specific T cells after increasing rounds of stimulation. Data shown are representative of one donor. (**g**) Gene expression of *KCNA3* and *KCNN4* in purified T cells after 1° or 4° TT stimulation. Expression of each channel is presented relative to expression in naive T cells. Data shown are representative of three donors. (**h**) Kv1.3 surface protein expression on 1° TT or 4° TT cells. Blue histogram represents Kv1.3 staining; grey filled histogram represents isotype control.

As described above, rat T cells become dependent on Kv1.3 following multiple rounds of antigen-specific stimulation. To explore whether human T cells are similarly driven from KCa3.1 towards Kv1.3 dependency through repeated antigen stimulation, we examined human T-cell responses to tetanus toxoid (TT) either directly *ex vivo* or after multiple rounds of *in vitro* stimulation. We selected TT as most donors have prior exposure to the antigen from vaccination against tetanus. *Ex vivo* T-cell responses to stimulation with TT were only weakly inhibited by ShK, but strongly inhibited by TRAM-34, with similar effects observed for CD4^+^ and CD8^+^ T cells (Fig. 4a–c). As observed previously in rat T cells, the combination of ShK and TRAM-34 fully abrogated T-cell responses (Fig. 4d). Expression of *KCNA3* and *KCNN4* by mRNA confirmed that these were the only two K^+^ channels expressed on these T cells *ex vivo* (Supplementary Fig. 5a,b). TT-specific T-cell lines were generated by multiple rounds of TT restimulation together with autologous APCs. After four rounds of stimulation, the sensitivity of TT-specific CD4^+^ T cells to ShK and TRAM-34 was reversed, with ShK inhibiting proliferation and IFN-γ production more profoundly than TRAM-34 (Fig. 4e). Sensitivity to ShK was increased with each round of stimulation while TRAM-34 sensitivity was reduced (Fig. 4f). Supporting the observed differential response to ShK and TRAM-34, expression of *KCNA3* and *KCNN4* by mRNA showed Kv1.3 was increased and KCa3.1 decreased in TT T cells after four rounds of stimulation relative to expression after the primary stimulation (Fig. 4g and Supplementary Fig. 5a,b). Concomitant increases in Kv1.3 cell surface protein expression were detected in repeatedly stimulated TT-specific T cells (Fig. 4h).

**Figure 5:**
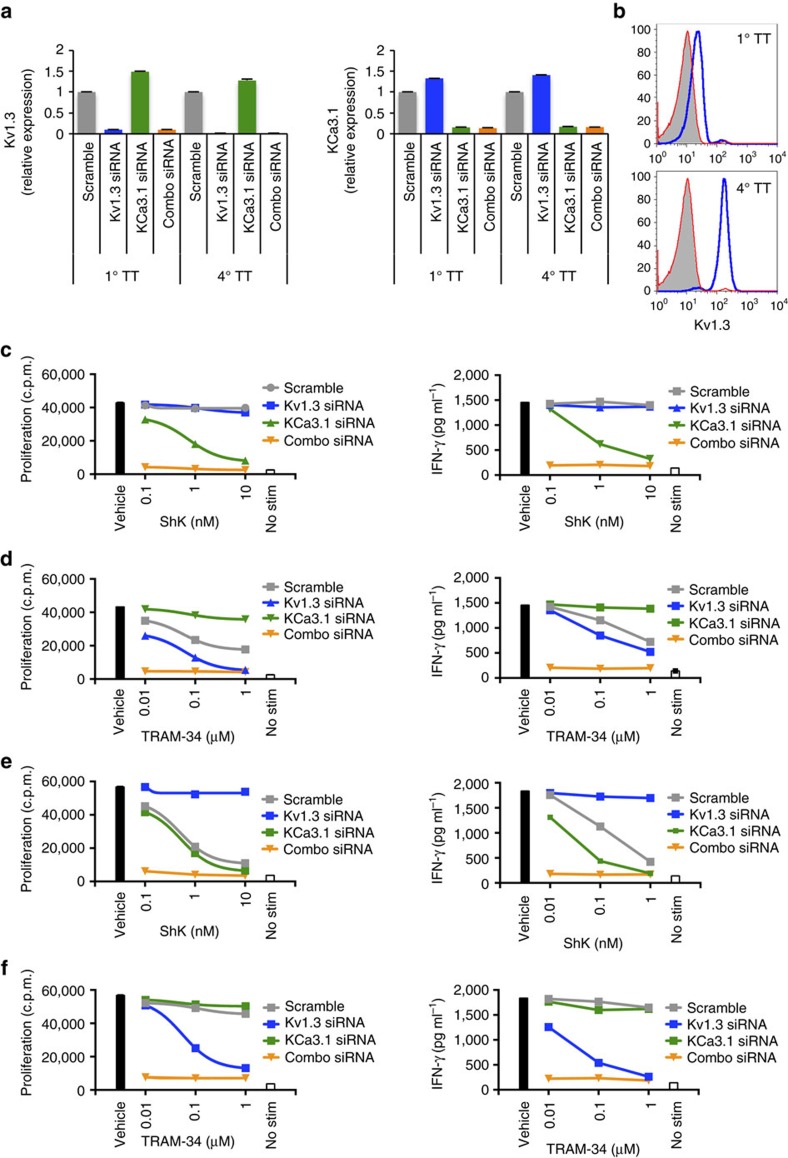
Effects of siRNA knockdown of Kv1.3 or KCa3.1 on TT-specific human T-cell sensitivity to inhibitors. (**a**) Kv1.3 (left) and KCa3.1 (right) expression was measured in primary TT-stimulated T cells (1° TT) or T cells that underwent four rounds of TT stimulation (4° TT) that were transfected with Kv1.3 siRNA (blue bars), KCa3.1 siRNA (green bars), a combination of both siRNA (orange bars) or scramble control siRNA (grey bars). Relative expression of targeted genes is shown in comparison with scramble siRNA transfected cells. Data are shown as mean±s.d. of triplicate measurements from one representative experiment. (**b**) Kv1.3 surface protein expression on 1° TT or 4° TT cells following siRNA transfection. Primary (left) or repeatedly stimulated (4°, right) TT cells were transfected with Kv1.3 siRNA (red histograms) or scramble siRNA (blue histograms) and then stained for Kv1.3 48 h later. Grey filled histogram represents isotype control. T-cell responses of 1° TT-stimulated T cells (**c**,**d**) or 4° TT-stimulated T cells (**e**,**f**) transfected with Kv1.3 siRNA (blue), KCa3.1 siRNA (green), a combination of both (orange) or scramble control siRNA (black). Cells were then restimulated with anti-CD3 (0.5 μg ml^−1^) in the absence or presence of ShK (**c**,**e**) or TRAM-34 (**d**,**f**) at the indicated concentrations. Proliferation responses were determined at day 4 of culture by ^3^H-thymidine incorporation; IFN-γ concentration was measured in culture supernatants harvested 3 days after restimulation. Data are shown as mean±s.d. of replicate wells and are representative of at least two independent experiments.

Despite the changes in expression, TT-specific cell lines required both Kv1.3 and KCa3.1 for maximal responsiveness. To confirm this and ask whether Kv1.3 was critical for T-cell activity, we performed siRNA-targeted knockdown of Kv1.3, KCa3.1 or both in the TT-specific recall responses *ex vivo* and in the TT-specific T cell-lines. Kv1.3 expression was reduced by at least 90% with Kv1.3 or combination siRNA and KCa3.1 expression reduced by about 85% (Fig. 5a). Knockdown of Kv1.3 protein expression was confirmed by flow cytometry (Fig. 5b). In primary TT-stimulated T cells, *KCNN4* siRNA knockdown conferred sensitivity to ShK and rendered these cells insensitive to TRAM-34, indicating that, while KCa3.1 is the predominant channel on antigen-specific T cells, Kv1.3 can functionally compensate for the loss KCa3.1 (Fig. 5c,d). Silencing of Kv1.3 enhanced sensitivity to TRAM-34, indicating that endogenous Kv1.3 functionally complements KCa3.1 (Fig. 5c,d). In TT-specific T-cell lines subjected to four rounds of stimulation, knockdown of Kv1.3 reversed the inhibitory effects of ShK and conferred sensitivity to TRAM-34, whereas knockdown of KCa3.1 had minimal impact (Fig. 5e,f). Disruption of both Kv1.3 and KCa3.1 expression rendered T cells unresponsive to *in vitro* recall stimulation, regardless of the number of exposures to antigen. Thus, in vaccine-induced memory T cells, Kv1.3 and KCa3.1 both have important roles in mediating T-cell responses.

### Autoreactive T cells are primarily dependent on Kv1.3

**Figure 6:**
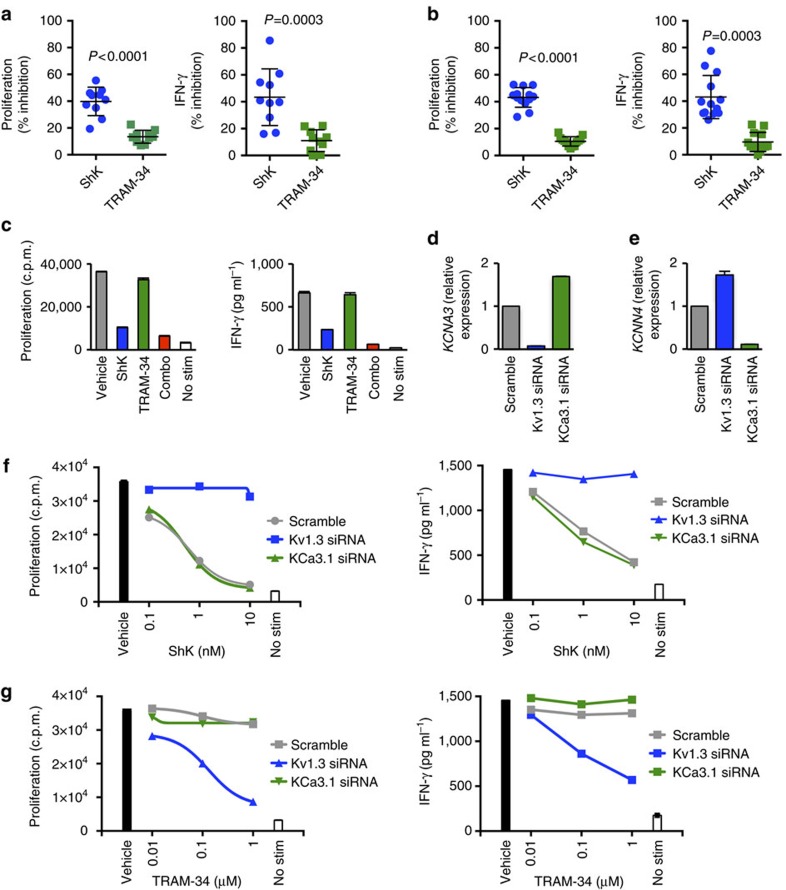
Kv1.3 and KCa3.1 are both required for human autoreactive T-cell responses. (**a**) PBMC from HLA-DR4^+^ T1D donors (*n*=10 biological replicates) were stimulated with a combination of HLA-DR4-restricted GAD65 peptides in the absence or presence of either 10 nM ShK or 1 μM TRAM-34. (**b**) PBMC from non-HLA- or HLA-typed T1D donors were stimulated with GAD65 protein (*n*=13 biological replicates). Proliferation responses (left) were determined at day 4. IFN-γ concentrations (right) were measured 3 days after stimulation. %inhibition was determined by comparing proliferation or IFN-γ concentration in the presence of inhibitor to vehicle control. Data are shown as individual data points with mean±s.d. Statistically significant differences are denoted with *P* values as determined by Student's *t-*test. (**c**) Inhibition of both Kv1.3 and KCa3.1 fully abrogates autologous autoreactive T cell responses. PBMC from T1D donors were stimulated with GAD65 protein in the absence or presence of 10 nM ShK, 1 μM TRAM-34 or a combination of both (combo). ‘No stim' denotes unstimulated cell conditions (absence of GAD65 protein). Data are shown as mean±s.d. of replicate wells and are representative of three independent experiments. Gene expression for Kv1.3 (*KCNA3*, **d**) and KCa3.1 (*KCNN4,* **e**) in GAD65-stimulated purified T cells following siRNA transfection with Kv1.3 siRNA (blue), KCa3.1 siRNA (green) or scramble control siRNA (grey). (**f**,**g**) Effects of siRNA knockdown of Kv1.3 or KCa3.1 on sensitivity to inhibitors. PBMC from T1D donor was stimulated with GAD65 protein for 7 days. After 3 days rest, T cells were purified and transfected with Kv1.3 siRNA (blue), KCa3.1 siRNA (green) or scramble control siRNA (grey) and then restimulated with anti-CD3 (0.5 μg ml^−1^) in the absence or presence of ShK (**f**) or TRAM-34 (**g**) at the indicated concentrations. Data are shown as mean±s.d. of replicate wells and are representative of at least two independent experiments.

Unlike vaccine-induced T cells, autoreactive T cells have been chronically exposed to their specific autoantigen, and therefore we asked if there was different K^+^ ion channel dependency in autoreactive T cells. Peripheral blood mononuclear cells (PBMCs) from HLA-typed Type I diabetes donors were stimulated with a pool of four HLA-DR4-restricted GAD65-derived peptides or with GAD65 protein in the presence of Kv1.3 inhibitor ShK or KCa3.1 inhibitor TRAM-34. ShK inhibited GAD65-specific T-cell proliferation and IFN-γ production, but the effect was only partial; in contrast, T-cell responses were minimally affected by TRAM-34 (Fig. 6a,b). Differential sensitivity to ShK and TRAM-34 was similar whether peptide pool or whole protein was used, and similar effects were observed on both CD4^+^ and CD8^+^ T cells (Supplementary Fig. 6b,c). Combined blockade of both Kv1.3 and KCa3.1 resulted in full inhibition of GAD65-specfic T-cell responses (Fig. 6c).

Complete inhibition was achieved when both ShK and TRAM-34 were present, suggesting that both Kv1.3 and KCa3.1 are functionally expressed. However, Kv1.3 is the predominantly active channel as seen by the greater susceptibility to ShK. To explore whether KCa3.1 is functional in GAD65-specific T cells, Kv1.3 expression was ablated using siRNA knockdown (Fig. 6d). Conversely, KCa3.1 was targeted (Fig. 6e) to determine its relative contribution to regulation of T-cell responses. siRNA knockdown of *KCNA3* reversed the inhibitory effects of ShK, rendering GAD65-specific T-cell proliferation and IFN-γ responses unaffected by ShK (Fig. 6f). Susceptibility to TRAM-34 was enhanced, however, indicating that in the absence of Kv1.3, KCa3.1 is present and sufficient to mediate T-cell activation (Fig. 6g). *KCNN4* siRNA knockdown had no effect on either ShK or TRAM-34 sensitivity (Fig. 6f,g), validating the notion that Kv1.3 is the predominant channel in GAD65-specific autoreactive T cells.

### Kv1.3/KCa3.1 dominance depends on antigen exposure history

**Figure 7:**
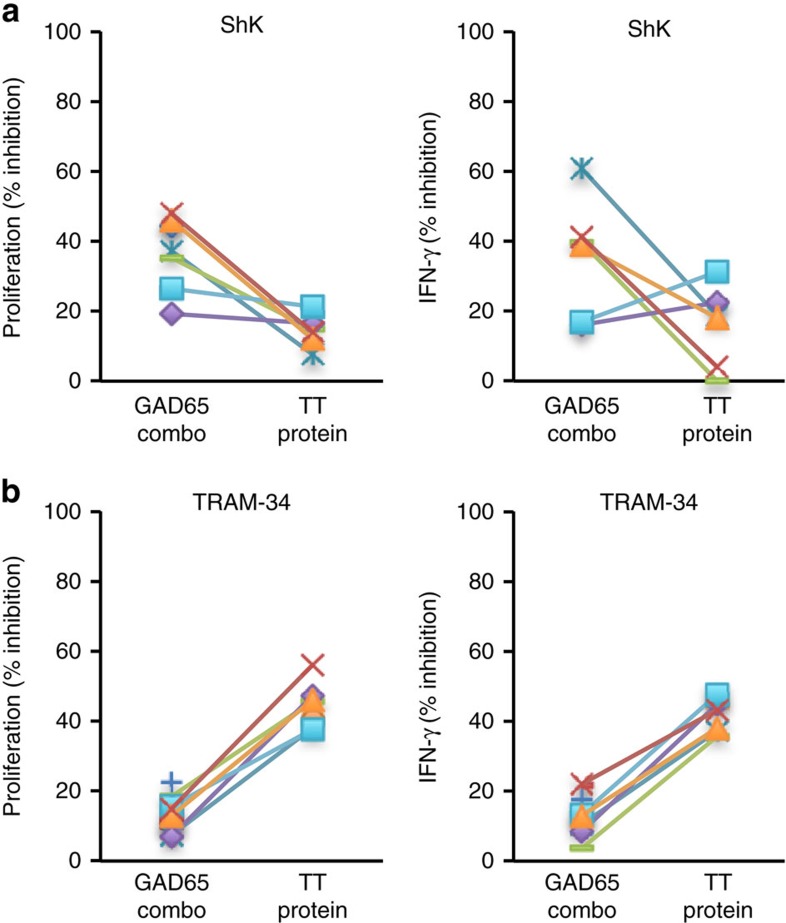
Autoreactive but not autologous pathogen-specific T cells are sensitive to Kv1.3 blockers. (**a**,**b**) ShK inhibits GAD65-specific T1D T cells but not autologous TT-specific T cells. PBMC from T1D donors (*n*=6 biological replicates) were stimulated with a combination of four HLA-DR4-restricted GAD65 peptides. Proliferation and IFN-γ responses were compared with TT stimulation in the absence or presence of either 10 nM ShK (**a**) or 1 μM TRAM-34 (**b**). Data shown are % inhibition of proliferation response as determined by ^3^H-thymidine incorporation after 4 days primary *in vitro* stimulation and % inhibition of IFN-γ production after 3 days stimulation. Coloured symbols and corresponding lines represent each individual donor.

Human T cells that have experienced chronic antigen-specific stimulation, either in the autoimmune setting or through repeated *in vitro* stimulation, were found to be preferentially dependent on Kv1.3. As the T-cell repertoire is diverse and reflects the history of antigen exposure experienced by an individual, we next examined whether T cells from the same donor, but with different antigen specificities, had differential biases for Kv1.3 or KCa3.1. PBMC from T1D donors were stimulated *in vitro* with either GAD65, representing an antigen recognized by chronically exposed T cells, or a pathogen-derived antigen such as TT, against which a resting memory pool of T cells is present. Comparing the GAD65-specific to TT-specific T-cell responses from the same donor, differential sensitivities to ShK and TRAM-34 were seen. Consistent with earlier data, GAD65-specific T-cell responses were inhibited by ShK (Fig. 7a), whereas TRAM-34 inhibited autologous TT-specific T-cell responses (Fig. 7b and Supplementary Fig. 6a,b). Influenza A hemagglutinin (HA)- and cytomegalovirus (CMV) -specific responses from T1D donors were also inhibited by TRAM-34 but not ShK (Supplementary Figs 7 and 8).

### Conversion to Kv1.3 dependency is stable

**Figure 8:**
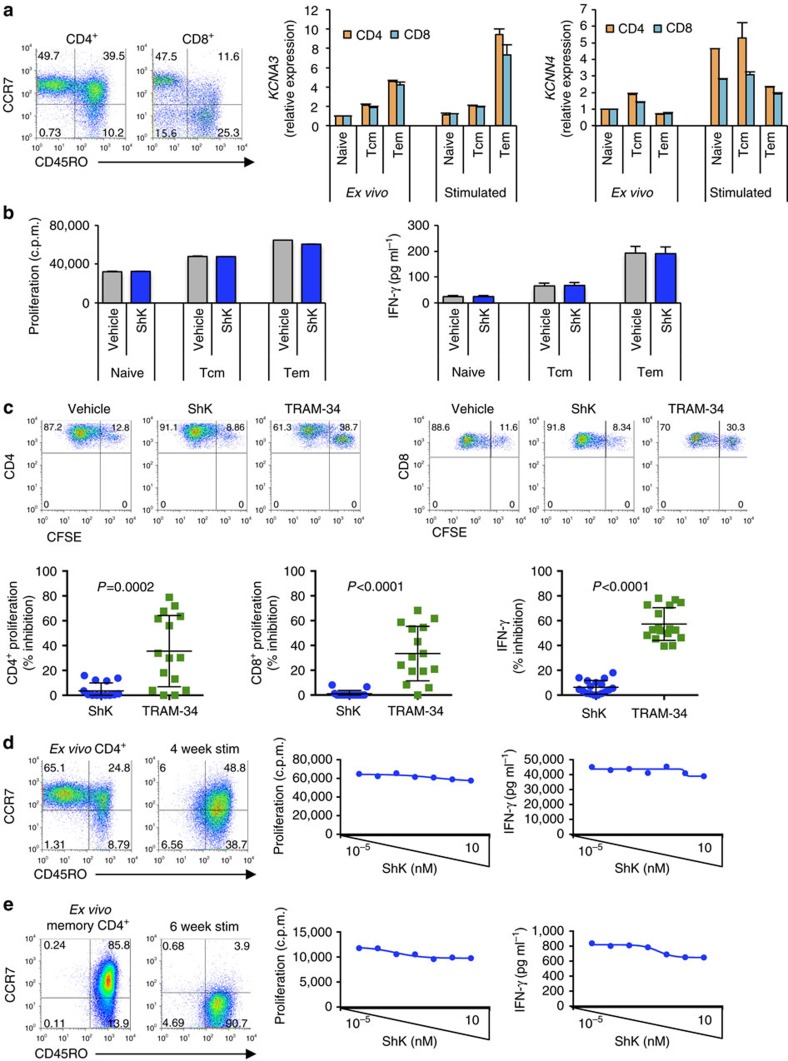
Effector memory T-cell phenotype is not sufficient to confer sensitivity to ShK-mediated inhibition. (**a**) Expression of Kv1.3 and KCa3.1 on sorted CD4^+^ and CD8^+^ naive, T_CM_ and T_EM_ subsets. CD45RO and CCR7 as markers to define naive, T_CM_ and T_EM_ subsets as shown in dot plots. Expression was determined on *ex vivo* cell subsets or on sorted T-cell subsets after 4 days stimulation with anti-CD3 and anti-CD28. Kv1.3 and KCa3.1 expression were normalized to RPL19 expression and shown relative to expression in *ex vivo* naive cells. (**b**) Effect of ShK on naive, T_CM_ and T_EM_ CD4^+^ subsets. Sorted cells were stimulated with anti-CD3 and either vehicle or 1 μM ShK. Proliferation was determined at day 4 of stimulation; IFN-γ was measured after 3 days. (**c**) Bulk PBMC from healthy donors were stimulated with anti-CD3 and anti-CD28 in the absence or presence of 10 nM ShK or 1 μM TRAM-34. IFN-γ production was measured after 3 days stimulation. Proliferation responses were determined at day 7 by CFSE dilution of CD4^+^- or CD8^+^-gated cells. Representative dot plots from one donor are shown. % inhibition of proliferation was determined by comparing proliferation in the presence of inhibitor to proliferation of cells in the presence of vehicle control. Data are shown as individual data points with mean±s.d. (*n*=15 biological replicates). (**d**) CD4^+^ T cells repeatedly stimulated with anti-CD3 and anti-CD28. Purified CD4^+^ T cells from healthy donor were stimulated with 5 μg ml^−1^ plate-bound anti-CD3 and 2 μg ml^−1^ soluble anti-CD28 for 4 days. Cells were then harvested, washed, and rested for 3 days. In subsequent stimulations, cells were reactivated with 1 μg ml^−1^ soluble anti-CD3 and 1 μg ml^−1^ anti-CD28. In the fourth round of stimulation, cells were cultured with 10-fold increases in ShK concentrations, starting at 10^*−*5^ nM. Cell supernatants were harvested 3 days after initiation of last round of stimulation for determination of IFN-γ. Proliferation responses were determined at day 4 by ^3^H-thymidine incorporation. (**e**) Purified memory CD4^+^ T cells were stimulated for six rounds with anti-CD3 and anti-CD28, as described in **d**.

KCa3.1 and Kv1.3 are both functionally expressed in all T cells, regardless of their antigen experience. However, in the initial exposure, KCa3.1 is the predominant channel that is involved in T-cell activation, as evidenced by higher relative gene expression levels and sensitivity to TRAM-34 inhibition. As T cells are repeatedly exposed to antigen, functional K^+^ channel requirement skews towards Kv1.3, as Kv1.3 relative gene expression is increased and T cells become susceptible to ShK. The conversion from KCa3.1 dependence to Kv1.3 driven by repeated antigen stimulation appears to be part of a progressive differentiation process in memory T cells. Notably, antigen-specific stimulation is a key requirement in this process, as T cells with the conventional CCR7^*−*^CD45RO^+^ T_EM_ phenotype are not affected by ShK when polyclonally activated with anti-CD3 and anti-CD28 antibodies (Fig. 8). This was demonstrated using sorted naive, T_EM_ and central memory (T_CM_) CD4^+^ and CD8^+^ T cells (Fig. 8a–c), purified CD4^+^ T cells repeatedly stimulated with anti-CD3 (Fig. 8d) and repeatedly anti-CD3 stimulated purified memory CD4^+^ T cells (Fig. 8e).

To address whether the shift to Kv1.3 with repeated antigen stimulation was stable, T cells that had undergone primary *in vitro* stimulation with TT in the absence or presence of ShK or TRAM-34 were rested, then restimulated. In the primary stimulation, T cells treated with ShK responded as well as vehicle control-treated cells, whereas responses were inhibited by TRAM-34 (Supplementary Fig. 9a). In the secondary stimulation, ShK and TRAM-34 treatment had the same effect on T cells regardless of their prior exposure to channel blockers. T cells treated with TRAM-34 had reduced proliferation and IFN-γ production in the primary stimulation, but were affected by ShK to a similar degree as T cells initially treated with vehicle control or ShK. This suggests that treatment with TRAM-34 did not influence K^+^ channel expression. These findings were consistent with TT-specific T cells that underwent further rounds of restimulation (Supplementary Fig. 9b). TT T cells having undergone three rounds of stimulation were sensitive to ShK. In the restimulation, T cells retained ShK sensitivity, indicating that prior blockade of Kv1.3 did not induce a shift to KCa3.1 (Supplementary Fig. 9c). Thus, prior exposure to inhibitor did not alter Kv1.3 or KCa3.1 channel dependence, suggesting that the K^+^ channel requirements are stable and are a function of antigen exposure history.

## Discussion

Electrophysiological patch-clamp methods allow specific channel activity to be studied at the single-cell level, and have thus been an invaluable tool contributing to the understanding of the role of Kv1.3 and KCa3.1 in purified, well-defined T-cell subtypes. The limitation of these methods, however, is that T-cell activation under physiological conditions is complex, and two channels cannot be studied simultaneously in the same cell. While exquisite control of voltage and membrane potential allows for study of Kv1.3 and Ca^2+^ influx for KCa3.1, these factors go hand-in-hand with TCR activation. Thus, while channel blockers can fully abrogate-specific channel function in patch-clamp experiments, we find that *in vitro* stimulated T-cell responses are only partially inhibited. Others have also observed partial inhibition with ShK and more selective/potent variants, or other Kv1.3 blockers, when functional readouts such as proliferation or cytokine production were used to assess T-cell responses, even at concentrations orders of magnitude greater than *K*_d_ values determined by electrophysiology^12^,^13^,^14^,^15^,^16^,^20^,^25^,^26^.

We generated a Kv1.3-deficient rat to definitively assess the requirement for Kv1.3 in elaboration of T-cell responses under various stimulation conditions. *Kcna3*^*−*/*−*^ rat T cells were fully functional when polyclonally activated or stimulated with specific antigen in *in vitro* recall assays, demonstrating that Kv1.3 was not required under these primary activation conditions. Using a CD4^+^ T-cell-dependent model of arthritis, *Kcna3*^*−*/*−*^ rats developed disease similarly to WT rats, indicating that Kv1.3 deficiency did not compromise immune CD4^+^ T-cell function. We then used DTH as a model where inflammation has been shown to be dependent on infiltrating T_EM_ cells that express high levels of Kv1.3 (refs 21, 32). DTH studies in *Kcna3*^*−*/*−*^ rats allowed for study of the effects of complete absence of Kv1.3 on generation of inflammatory responses. Ear inflammation in *Kcna3*^*−*/*−*^ rats was not dramatically different from WT, with one experiment showing slightly reduced inflammation but a second having mildly more severe inflammation. Thus, in the complete absence of Kv1.3, T cells were uncompromised in their ability to perform effector functions and mediate inflammation, likely due to the presence of KCa3.1. Our DTH studies in *Kcna3*^*−*/*−*^ rats were consistent with the modest therapeutic effects achieved through pharmacological blockade of Kv1.3. Previous reports have shown that efficacy could be achieved with Kv1.3 inhibitors, but highly variable effects were observed, with reduction ranging from no significant effect to as high as 80%, with various compounds^12^,^13^,^15^,^25^,^26^,^33^,^34^,^35^. The complementary and compensatory roles of Kv1.3 and KCa3.1 in T cells is also supported by published studies where treatment with TRAM-34 to block KCa3.1 channels ameliorated inflammation in DTH^36^. In addition, while our studies have focused on the K^+^ channels, other channel types such as store-operated Ca^2+^ entry channel CRAC and non-store-operated Ca^2+^ channel TRPV1 also have roles in TCR-induced Ca^2+^ influx and regulation of TCR signalling and T-cell activation^37^,^38^.

In addition to demonstrating that Kv1.3 was not required to mediate a T_EM_-dependent inflammatory response, the DTH model revealed that repeated OVA immunization increased T-cell susceptibility to Kv1.3 inhibition. T cells from rats receiving a single primary immunization, T-cell proliferation and cytokine production were inhibited by TRAM-34 and are relatively insensitive to ShK treatment. Conversely, T cells from rats repeatedly immunized with OVA exhibited increased sensitivity to ShK associated with an increase in Kv1.3 expression.

Importantly, we also observed that the inhibition mediated by single-channel blockers was not complete, and a combination of both ShK and TRAM-34 was required to achieve full abrogation of T-cell responses. These findings indicate that Kv1.3 and KCa3.1 are sufficient to regulate the T-cell response, and suggest that other K^+^ channels such as the two-pore domain K^+^ channel TASK1 (refs 39, 40) do not play a significant role. This validated our findings in the *Kcna3*^*−*/*−*^ rat, showing that KCa3.1 was sufficient to mediate normal activation responses in T cells normally reliant on Kv1.3 and that KCa3.1 may compensate for impaired Kv1.3 function mediated by blockers such as ShK. Repeated antigen stimulation conferred a functional Kv1.3 expression phenotype that is consistent with published data using repeatedly activated human autoreactive T cells. CCR7 expression was reduced in T cells isolated from animals chronically exposed to antigen, indicating that T cells were differentiating towards a T_EM_ phenotype. That sensitivity to ShK was elevated in cells having reduced CCR7 expression is consistent with the notion that autoreactive T_EM_ cells would be amenable to Kv1.3 blockade.

On the basis of the rat data, susceptibility to Kv1.3 inhibitor is a property of repeatedly stimulated antigen-specific T cells, with increasing exposures to antigen driving T-cell dependency on KCa3.1 towards Kv1.3 as the prevalent channel. The transition from ShK-insensitivity of primary stimulated antigen-specific T cells to ShK susceptibility following multiple rounds of stimulation suggests that antigen-specific T cells become ‘programmed' towards Kv1.3 dependency, and that this transition is not well demarcated but becomes enforced with chronic stimulation. Our data supports published reports that T cells isolated from patients with autoimmune disease, including multiple sclerosis, rheumatoid arthritis and Type 1 diabetes (T1D), express high levels of Kv1.3 on a per cell basis^11^,^15^,^16^,^20^,^41^. However, demonstration of autoreactive T-cell susceptibility to Kv1.3-targeted inhibition typically requires restimulation with antigen for as many as a dozen times^15^,^16^,^34^. Thus, the requirement for chronic antigen stimulation that we observed for rat T cells may be translatable to autoreactive human T cells.

We report that human T-cell dependency on Kv1.3 was also driven by repeated antigen stimulation. Using TT, CMV and influenza as model pathogens, we demonstrated that in a primary *in vitro* stimulation response, T-cell proliferation and cytokine production were inhibited by TRAM-34 and were relatively insensitive to ShK treatment. Conversely, T cells from T1D patients stimulated with an autoantigen such as GAD65 were sensitive to ShK, but not TRAM-34. Notably, repeated stimulation with TT increased sensitivity of antigen-specific T cells to ShK, conferring upon these cells a functional Kv1.3 expression phenotype similar to autoreactive T cells. We also observed that the inhibition mediated by ShK or TRAM-34 was not complete under these conditions, and a combination of both was required to achieve full abrogation of T-cell responses. Additionally, silencing of Kv1.3 expression in GAD65-specific and repeated stimulated TT-specific T cells rendered these cells insensitive to ShK, but surprisingly these cells were now sensitive to TRAM-34, indicating that functional KCa3.1 channels were present and active, compensating for the absence of Kv1.3.

Patch-clamp experiments have formed the basis for the Kv1.3/T_EM_ vs. KCa3.1/naive/T_CM_ expression paradigm. However, the conventional definition of T_EM_ cells using surface markers such as CCR7 and CD45RO may not be sufficient to identify those cells that have high functional levels of Kv1.3 expression and are thus more susceptible to Kv1.3 blockade. Antigen-specific T cells are comprised of a mix of T_EM_ and T_CM_ cells on the basis of CCR7 and CD45RO expression, and CD4^+^ T cells acquire the T_EM_ phenotype following repeated stimulation. However, T cells activated *in vitro* in the primary stimulation with specific pathogen-derived antigens are not susceptible to Kv1.3 inhibitors such as ShK. Nor are T cells driven to the CCR7^*−*^CD45RO^+^ phenotype through repeated anti-CD3 stimulation, which remain refractory to ShK inhibition. T cells derived from autoimmune disease patients stimulated with autoantigen have a similar mixed CCR7/CD45RO expression profile as pathogen-stimulated T cells, yet autoreactive T cells are amenable to Kv1.3 inhibitors. Of note, Kv1.3-targeted inhibition of T_EM_ cells in the literature is typically demonstrated on antigen-specific human or rat T-cell lines that have been restimulated with antigen for as many as 12 times^15^,^16^,^34^. Thus, a CCR7^+^CD45RO^+^ T_EM_ phenotype alone may not necessarily mark the T cell as a target for Kv1.3 inhibition.

On the basis of our combined data from rat and human T cells, susceptibility to Kv1.3 inhibitor is a property of repeatedly stimulated antigen-specific T cells. Activation alone is not sufficient since repeated anti-CD3 stimulation does not increase sensitivity to ShK; instead, it is the number of exposures to antigen that dictates whether Kv1.3 or KCa3.1 is the prevalent channel. The transition from ShK-insensitivity of primary stimulated TT-specific T cells to ShK susceptibility following multiple rounds of stimulation suggested that antigen-specific T cells become ‘programmed' towards Kv1.3 dependency, and that this transition was not well demarcated but became enforced with chronic stimulation. Repetitive antigen stimulation is a factor that can influence memory T-cell differentiation. For CD8 T cells, multiple antigen encounters, through prime-boost vaccinations or pathogen reencounter, increase memory T-cell numbers and dramatically impact their phenotype and function^42^,^43^,^44^,^45^. Several hundred genes can potentially be differentially regulated with each subsequent round of antigen encounter, resulting in memory cells with unique transcriptome repertoires^46^. The mechanisms responsible for conversion from KCa3.1 to Kv1.3 dependency remain to be elucidated.

Collectively, we demonstrate that Kv1.3 is the predominant functionally active channel in T cells subjected to repeated specific antigen exposure and that KCa3.1 compensates for loss of functional Kv1.3 as in the presence of Kv1.3 inhibitors. Prior reports of differential expression of Kv1.3 and KCa3.1 on discrete subsets of T cells and their specific inhibition by selective inhibitors have made these K^+^ channels attractive therapeutic targets. In particular, the suggested predominance of Kv1.3 on T_EM_ cells has led to profound interest in developing Kv1.3 inhibitors for suppression of autoimmune reactions. Our work supports the possibility of achieving beneficial effects by targeting Kv1.3 in the autoimmune context, but with the caveat that the inhibition of autoreactive T-cell functions may not be complete due to compensatory actions of KCa3.1.

## Methods

### *Kcna3* ^*−*/*−*^ rats

*Kcna3*^*−*/*−*^ rats were generated on Dark Agouti background at SAGE Labs (Boyertown, PA). Zinc finger nuclease targeting produced an 1178, bp deletion in *Kcna3* corresponding to genomic coordinates chr2:229,233,225-229,234,403 (m5 genome assembly). *Kcna3*^*−*/*−*^ and WT littermates were housed and maintained at Genentech in accordance with American Association of Laboratory Animal Care guidelines. Eight- to ten-week-old female rats were used in all experiments. All experimental animal studies were conducted under the approval of the Institutional Animal Care and Use Committees of Genentech Lab Animal Research.

### Human cells

Human PBMC were isolated from donor blood by Ficoll gradient centrifugation. Blood from healthy control and CMV^+^ donors was collected as part of the Genentech blood donor program, with written informed consent and approval from the Western Institutional Review Board. T1D patient blood was obtained from BioreclamationIVT (non-HLA-typed, Westbury, NY), and HLA-DR0401-typed T1D blood from Benaroya Research Institute (Seattle, WA).

### Electrophysiology

Patch-clamp experiments were performed in whole-cell configuration using a SyncroPatch 768PE system (Nanion Technologies, Germany). Due to the relatively small size of T cells, single-hole, high-resistance (∼10 MΩ) borosilicate glass planar chips were used. Data was acquired with SyncroPatch PatchControl384 1.4.1 software and data analysis was performed with IgroPro 6.3.7.2 (Wavemetrics, OR) and Prism 6 (GraphPad, CA). The intracellular solution used contained (in mM): 50 KCl, 60 KF, 10 NaCl, 20 EGTA and 10 HEPES (pH 7.2, osmolarity 285 mOsm), and extracellular solution contained (in mM): 140 NaCl, 4 KCl, 2 CaCl_2_, 1 MgCl_2_, 5 Glucose and 10 HEPES (pH 7.4, osmolarity 300 mOsm). The holding potential for all experiments were set at −80 mV, and currents were elicited by depolarizing voltage steps from −60 to +40 mV (10 mV increments) for kinetic study or by repetitive pulses to 40 mV for all other studies. Currents were sampled at 20 kHz and filtered with Bessel filter. Series resistance was compensated 80% with leak subtraction. Seal resistance (Rseal) was calculated using built-in protocols, and cells with Rseal<500 MΩ were excluded from analysis. Kv1.3 channel numbers per cell were determined by dividing the 1 nM Shk-sensitive whole-cell conductance by the single-channel conductance of Kv1.3 (11 pS) (ref. 47). In pharmacological test, current was elicited every 30 s, 5 min for both before and after applying compound. Patch-clamp measurements are presented as the means±s.e.m.

### *In vitro* cell T-cell assays

Rat T-cell responses to anti-CD3 and anti-CD28 activation were determined using spleen cells from naive animals. 1 × 10^6^ spleen cells were plated on 96-well plates precoated with 5 μg ml^−1^ anti-rat CD3 (clone G4.18, BD Biosciences) in a final volume of 200 μl complete RPMI media (RPMI 1640 media supplemented with 10% FBS, 2 mM glutamine, 2 μM 2-ME, 1 mM sodium pyruvate, 100 U ml^−1^ penicillin and 100 μg ml^−1^ streptomycin) supplemented with 2 μg ml^−1^ soluble anti-rat CD28 (clone JJ319, BD Biosciences).

*In vitro* recall responses to OVA were performed using spleen and draining lymph node cells harvested from rats immunized with trinitrophenyl-OVA (TNP-OVA), with cells harvested 10 days post immunization. For *in vitro* recall responses to multiple antigens, rats were immunized at day 0 with TNP-OVA in CFA at 1 mg ml^−1^, followed by subsequent immunizations at day 14 and day 28 with TNP-OVA in incomplete Freund's adjuvant at 1 mg ml^−1^. At day 28, the rats were additionally immunized with MBP 68-86 peptide in incomplete Freund's adjuvant at 1 mg ml^−1^. Cells were harvested at day 38. For antigen-specific *in vitro* stimulation, DLN cells were plated at 1 × 10^5^ cells per well together with spleen cells at 1 × 10^6^ cells per well in final volume of 200 μl per well in 96-well plates in complete RPMI media. Cells were stimulated with OVA or MBP at 10 μg ml^−1^ unless otherwise indicated.

For assessment of APC function, CD4^+^ or CD8^+^ T cells were purified from draining lymph nodes and spleen of OVA-immunized WT and KO rats using magnetic bead separation (Miltenyi Biotec). Purity of isolated cells was validated using flow cytometry and was >95% for all cell types. For isolation of APCs, spleen cells were additionally depleted of T cells and 1 × 10^6^ T-cell-depleted spleen cells were plated and placed in 37° humidified incubator. After 1 h, nonadherent cells were gently aspirated off to leave adherent APCs. 1 × 10^5^ CD4^+^ or CD8^+^ T cells were plated in final volume of 200 μl complete RPMI and OVA added at 10 μg ml^−1^.

For rat T-cell assays, supernatants were harvested after 3-day culture for quantization of IFN-γ concentrations using rat IFN-γ Quantikine ELISA kit (Kit RIF100, R&D Systems). Proliferation was assessed using CellTiter-Glo (Promega), measuring relative luminescence units following manufacturer's protocol.

Antigen-specific *in vitro* cell stimulation of human cells was performed using TT (derived from *Clostridium tetani*, Calbiochem, EMD Millipore, Billerica, MA or Reagent Proteins, San Diego, CA), a combination of CMV pp65 PepMix (pool of 138 peptides derived from peptide scan through pp65 of human CMV) and IE-1 PepMix (pool of 120 peptides derived from immediate-early protein 1 of human CMV) (JPT Peptide Technolologies, Berlin, Germany), HLA-DR0401-restricted influenza-derived HA_306-318_ peptide (Benaroya Research Institute), GAD65 protein (Abcam, Cambridge, MA) or a combination of HLA-DR0401-restricted GAD65 P15, GAD65_265-284_, GAD65_273-292_ and GAD65_553-572_ peptides representing frequently recognized epitopes^48^ (Benaroya Research Institute). TT and GAD65 protein stimulations were performed at 5–10 μg ml^−1^, CMV PepMix combination stimulation was performed using 5 μg ml^−1^ of each PepMix, individual GAD65 peptide and HA peptide stimulations were performed at 10 and 2.5 μg ml^−1^ of each individual GAD65 peptide was used in the combination stimulation. Primary stimulations were cultured for 4–7 days, depending on the readout for proliferation. For repeated stimulations, cells were collected, spun down, washed, then rested for 3 days. For antigen-specific restimulation, frozen autologous PBMC were thawed and irradiated at 2000, rad, then plated with antigen and rested cells. This cycle was repeated for each round of restimulation. Cells were cultured in either complete RPMI media or in RPMI 1640 supplemented with 2 mM L-glutamine, pen/strep and 10% pooled human serum (MP Biomedicals, Santa Ana, CA). In restimulations, media was supplemented with human IL-2T cell growth factor (Hemagen Diagnostics, Columbia, MD).

For anti-CD3 stimulation of human T cells, primary activation was performed using 5 μg ml^−1^ plate-bound anti-human CD3 (clone SP34-2, BD Biosciences). A total of 2 μg ml^−1^ soluble anti-human CD28 (clone CD28.2, BD Biosciences) was added for primary activation when indicated. For subsequent restimulations, after 4 days activation, cells were rested for 3 days, then restimulated with 0.5–1 μg ml^−1^ anti-CD3.

Kv1.3-specific channel blocker ShK was purchased from Bachem (Torrance, CA) and resuspended in PBS. KCa3.1-specific blocker TRAM-34 was purchased from Sigma-Aldrich (St. Louis, MO) and resuspended in DMSO. Cells were preincubated with inhibitors for 30 min at 37 °C in humidified, 5% CO_2_ incubator, before stimulation.

In human T-cell assays, for carboxyfluorescein succinimidyl ester (CFSE) dilution, cells were labelled with 2.5 μM CFSE for 5 min at room temperature followed by extensive washing, before setting up stimulations. CFSE dilution was assessed by flow cytometry after 7 days. For [^3^H]-thymidine incorporation proliferation assays, 1 μCi [^3^H]-thymidine (Perkin-Elmer) in a volume of 50 μl was added to each well for the past 16–18 h of a 4 day culture. Cells were then harvested and [^3^H]-thymidine incorporation was measured by liquid scintillation counting. IFN-γ concentrations were measured in human cell culture supernatants using ELISA kits specific for human IFN-γ (BD Biosciences or R&D Systems).

Proliferation and IFN-γ data are expressed as either raw data values to show relative strength of responses, as percentages of vehicle-treated control values to allow normalization of inhibitor effects to responses in the absence of inhibitor, or conversion to per cent inhibition.

### Flow cytometry

Abs against rat antigens used for staining were all purchased from BD Biosciences: FITC-conjugated CD3 (clone G4.18); PE-conjugated CD25 (clone OX-39), CD45R (clone HIS24), CD45RC (clone OX-22); PerCP-conjugated CD8 (clone OX-8); PE-Cy5-conjugated CD45RA (OX-33); APC-conjugated CD3 (clone 1F4), CD4 (clone OX-35). Abs against human antigens used for staining were all purchased from BD Biosciences: PE-conjugated CD4 (clone RPA-T4), CCR7 (clone 150503); PerCP-conjugated CD4 (clone SK3), CD8 (Clone SK1); APC-conjugated CD25 (clone M-A251), CD45RO (clone UCHL1). FITC-conjugated Kv1.3 Ab was purchased from Sigma-Aldrich. Samples were acquired on a FACSCalibur flow cytometer using CellQuest Pro v5.1.1 software (BD Biosciences) and data analysis performed using FlowJo v6.4.2 software (Tree Star, Inc.). Cell sorting was performed on FACSAria to isolate purified populations of human naive (CCR7^+^CD45RO^*−*^), T_CM_ (CCR7^+^CD45RO^+^) and T_EM_ (CCR7^*−*^CD45RO^+^) CD4^+^ or CD8^+^ subsets. In some instances CD4^+^ and memory CD4^+^ T cells were purified using appropriate MACS cell isolation kits (Miltenyi Biotec). Purity of isolated cells was validated using flow cytometry and was >95% for all cell types. General gating strategy for CD4+ and CD8+ T cells is shown in Supplementary Fig. 6a.

### Adjuvant-induced arthritis

Seven- to eight-week-old Kv1.3 KO and WT rats received a single injection of 100 μl complete Freund's adjuvant at the base of the tail at 1 mg ml^−1^. Animals were monitored daily and visual scoring for each paw was assessed as follows: 0=no evidence of erythema and swelling; 1=erythema and mild swelling confined to the mid-foot (tarsal) or ankle; 2=erythema and mild swelling extending from the ankle to the mid-foot; 3=erythema and moderate swelling extending from the ankle to the metatarsal joints; 4=erythema and severe swelling encompass the ankle, foot and digits. Mean score=sum of the four paw scores. Disease stages: mild (mean score 1–3), moderate (mean score 4–8) and severe disease (mean score>9).

### Delayed-type hypersensitivity response

Seven- to eight-week-old Kv1.3 KO and WT littermate rats were immunized at the base of the tail with 200 μl emulsion containing 100 μg TNP-OVA dissolved in 100 μl PBS mixed with 100 μl of CFA at 1 mg ml^−1^. On day 7, right pinna ear thickness was measured, and then challenged with injection of 25 μl PBS alone or TNP-OVA dissolved in PBS at 2 mg ml^−1^. On day 8, right pinna ears were again measured. Delta ear thickness is the thickness of post-challenge measurement subtracting pre-challenge measurement. For studies where treatment was administered, CTLA4-Fc or anti-ragweed was administered at 6 mg kg^−1^ in PBS delivered three times a week via intraperitoneal route starting on the day before immunization. The generation, production and purification of CTLA4-Fc and anti-ragweed antibodies used in this study has been described elsewhere^49^. ShK peptide was administered at 200 μg kg^−1^ on days 6 and 7, before challenge and during challenge, respectively. ShK was delivered in PBS via intravenous route.

### RNA isolation and quantitative RT-PCR

For analysis of gene expression, RNA was isolated from cells using RNeasy Mini Kit (Qiagen). TaqMan real-time quantitative RT-PCR was performed using the ABI7500 Real-Time PCR system (Applied Biosystems). Rat TaqMan Gene Expression Assay primer/probe sets were from Applied Biosystems: *Kcna3* (Rn00570552_s1), *Kcnn4* (Rn00576373_m1), *Kcna1* (Rn00597355_s1), *Kcna2* (Rn02769834_s1), *Kcna4* (Rn02532059_s1), *Kcna5* (Rn00564245_s1), *Kcna6* (Rn01492950_s1), *Kcna7* (Rn01476090_m1), *Ccr7* (Rn01465443_m1). Human TaqMan Gene Expression Assay primer/probe sets were from Applied Biosystems: *KCNA3* (Kv1.3, Hs00704943_s1), *KCNN4* (KCa3.1, Hs00158470_m1), *KCNA1* (Kv1.1, Hs00264798_s1), *KCNA2* (Kv1.2, Hs00270656_s1), *KCNA4* (Kv1.4, Hs00937357_s1), *KCNA5* (Kv1.5, Hs00266898), *KCNA6* (Kv1.6, Hs00266903_s1), *KCNA7* (Kv1.7, Hs00361015_m1), *CCR7* (Hs01013469_m1). Mouse TaqMan Gene Expression Assay primer/probe sets were from Applied Biosystems: *Kcna3* (Mm00434599_s1), *Kcnn4* (Mm00464586_m1), *Kcna1* (Mm00439977_s1), *Kcna2* (Mm00434584_s1), *Kcna4* (Mm00445241_s1), *Kcna5* (Mm00524346_s1), *Kcna6* (Mm00496625_s1), *Kcna7* (Mm01197268_m1). Expression of ribosomal protein L19 was used to normalize copy number. Relative expression levels (fold differences) between groups were determined by ΔΔCt method.

### Small interfering RNA knockdown

siRNA knockdown of Kv1.3 and KCa3.1 expression was performed using ON-TARGETplus SMARTpool siRNA (KCNA3, L-006213-00-0005; KCNN4, L-004461-00-0005; negative control pool, D-001810-10) from Thermo Scientific. Cell populations were transfected with siRNA (300 nM final concentration) using Human T-cell Nucleofector Kit (Amaxa) according to manufacturer's recommended protocol.

### Statistics

Statistical analyses were performed using JMP version 9.0.2 software (SAS Institute). We made comparisons for each pair with Student's *t*-test, with *P* values <0.05 considered significant.

### Data availability

The data that support the findings of this study are available from the corresponding author upon request.

## Additional information

**How to cite this article:** Chiang, E. Y. *et al*. Potassium channels Kv1.3 and KCa3.1 cooperatively and compensatorily regulate antigen-specific memory T cell functions. *Nat. Commun.* **8,** 14644 doi: 10.1038/ncomms14644 (2017).

**Publisher's note:** Springer Nature remains neutral with regard to jurisdictional claims in published maps and institutional affiliations.

## Supplementary Material

Supplementary Information Supplementary Figures 1-9
