# Supplementary material for: Potassium channels Kv1.3 and KCa3.1 cooperatively and compensatorily regulate antigen-specific memory T cell functions
Source: Nat Commun. 2017 Mar 1;8:14644. doi: 10.1038/ncomms14644 (PMC5337993; doi:10.1038/ncomms14644)
Supplement: Supplementary Information — Supplementary Figures 1-9 [file ncomms14644-s1.pdf]

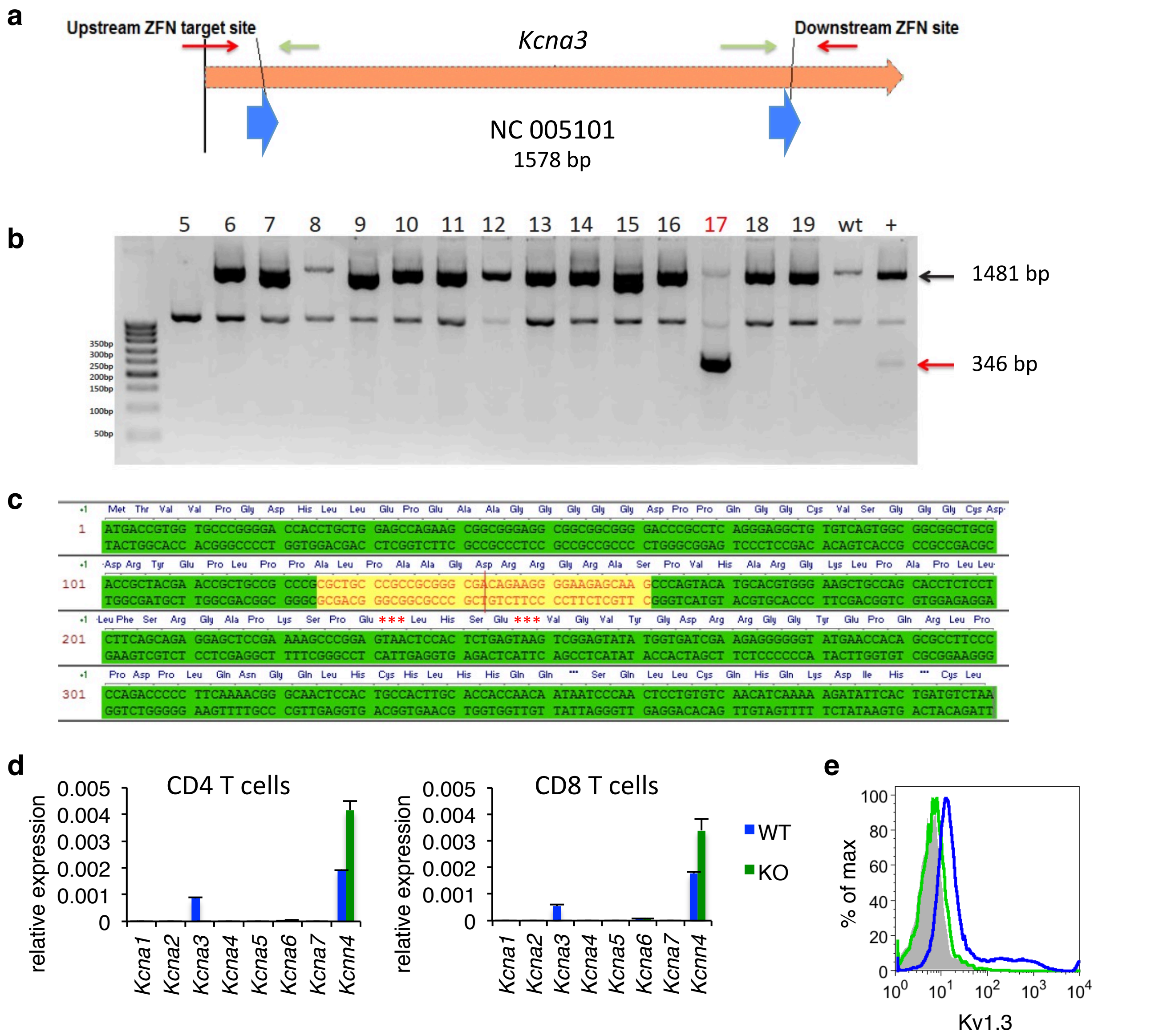

Supplementary Figure 1. Generation of *Kcna3*$^{-/-}$ rats. (**a**) Zinc finger nuclease (ZFN) strategy for deletion of *Kcna3* gene. Kcna3 gene consists of single exon, so two pairs of ZFNs were designed to cleave together in order to generate an approximately 1.2 kb deletion between the cut sites (NCBI genomic coordinates 144-1321 in reference sequence NC_005101). Primers flanking each ZFN site were designed to test individual NHEJ activity (green arrows) as well as paired together (red arrows) to screen for deletion mutations between the two ZFN sites. (**b**) Deletion screen to identify *Kcna3*$^{-/-}$ founder rats. Expected wild-type band = 1481 bp, expected deletion between ZFNs = 346 bp. (**c**) Sequence confirmation of *Kcna3* deletion in rat #17. ZFN targeted deletion of *Kcna3* resulted in 2 stop codons, indicated by red asterisks. (**d**) K^+ channel expression in WT and *Kcna3*$^{-/-}$ CD4$^+$ (*left panel*) and CD8$^+$ (*right panel*) T cells. Expression of Kv1.3 (*Kcna3*), other Kv1 family members (*Kcnax*), and KCa3.1 (*Kcnn4*) was measured in *ex vivo* purified T cells. Relative gene expression was determined by normalizing to housekeeping gene *Rpl19*. Data are shown as mean ± s.d. (e) Kv1.3 surface protein expression is absent on *Kcna3*$^{-/-}$ rat CD4$^+$ T cells. Blue histogram represents Kv1.3 expression on WT CD4$^+$ T cells, red histogram denotes KO; gray filled histogram represents staining with isotype control antibody.

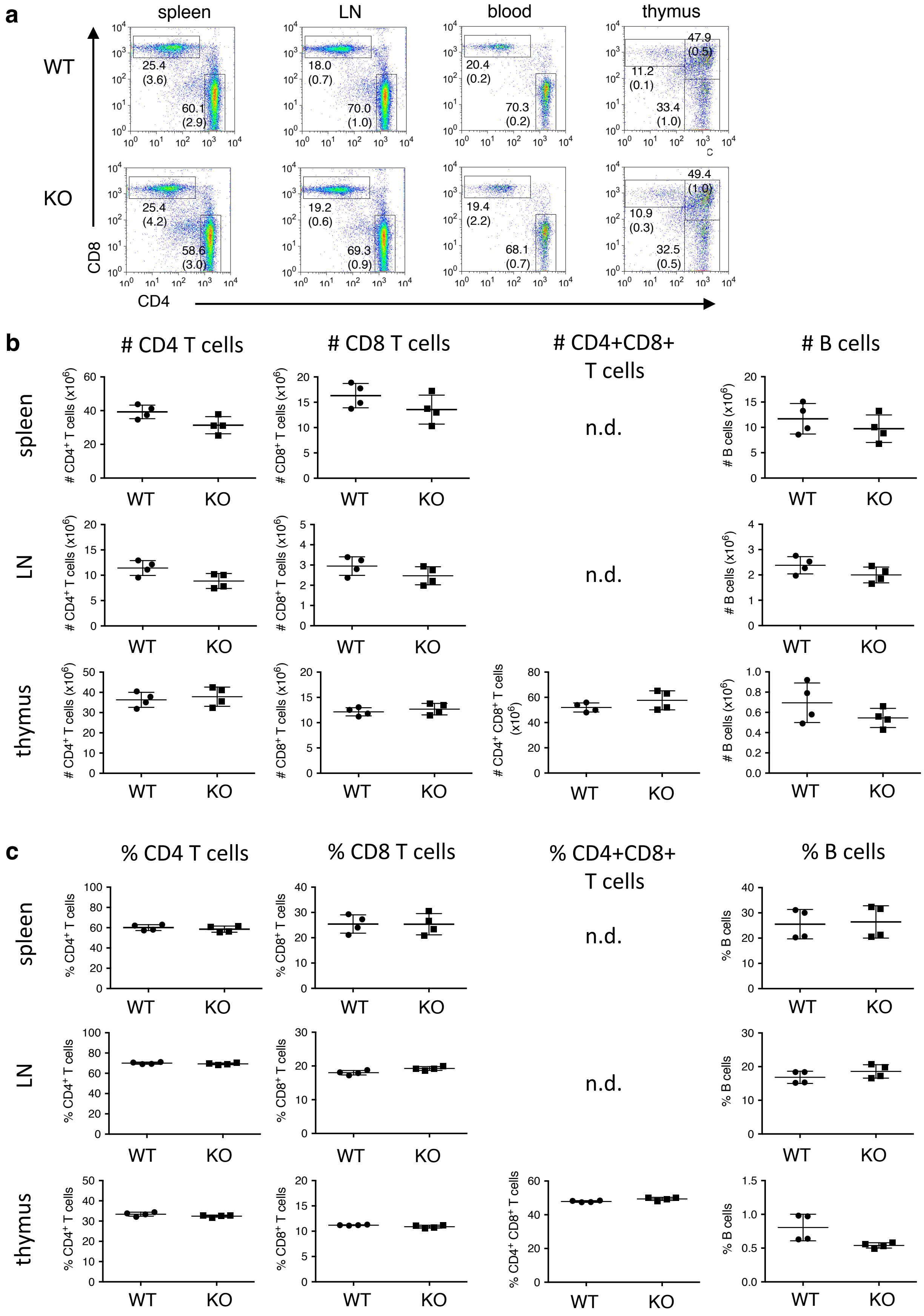
a
spleen
LN
blood
thymus
WT
KO
CD8
CD4
25.4 (3.6)
60.1 (2.9)
18.0 (0.7)
70.0 (1.0)
20.4 (0.2)
70.3 (0.2)
47.9 (0.5)
11.2 (0.1)
33.4 (1.0)
25.4 (4.2)
58.6 (3.0)
19.2 (0.6)
69.3 (0.9)
19.4 (2.2)
68.1 (0.7)
49.4 (1.0)
10.9 (0.3)
32.5 (0.5)
b
CD4 T cells
CD8 T cells
CD4+CD8+ T cells
B cells
spleen
LN
thymus
n.d.
WT
KO
c
% CD4 T cells
% CD8 T cells
% CD4+CD8+ T cells
% B cells

Supplementary Figure 2. Immune cell distribution in *Kcna3*$^{-/-}$ rats. **(a)** T cell frequencies in *Kcna3*$^{-/-}$ rats. Flow cytometry staining was used to define CD4^{+} and CD8^{+} T cell populations from spleen, pooled LN, blood and thymus. Data are shown for live CD3^{+} gated cells. Data are representative of 4 individual *Kcna3*$^{-/-}$ and WT littermate rats. Frequencies of specific cell populations are shown for indicated gated population as mean ± s.d. Flow cytometric analysis was used to enumerate T cell and B cell populations (**b**) or determine relative cell frequencies (**c**) in spleen, LN and thymus WT and *Kcna3*$^{-/-}$ rats. Individual animals (n = 4 biological replicates per group) are represented by discrete symbols and are shown with mean ± s.d.. No statistically significant differences as determined by Student's *t* test were observed between WT and KO for any cell population. "n.d." denotes cell subset not determined.

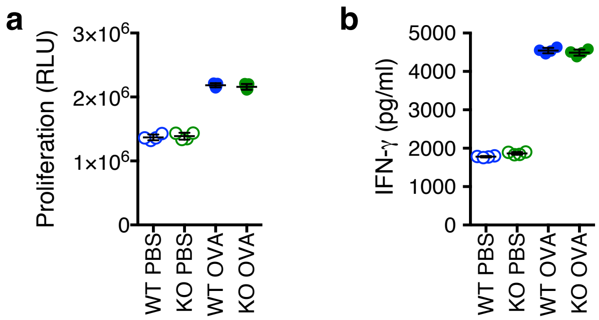

Supplementary Figure 3. OVA-specific *in vitro* recall responses from DTH rats. WT (*blue*) or *Kcna3*$^{-/-}$ (*green*) OVA-immunized rats were subsequently challenged with OVA (*filled symbols*) or PBS (*open symbols*). Proliferation (**a**) and IFN-γ (**b**) responses of DLN and spleen cells (1:10 ratio) harvested from animals receiving secondary challenge with PBS or OVA were determined after 3-day stimulation with OVA. Individual biological replicates (n = 4 per group) are shown with mean ± s.d.

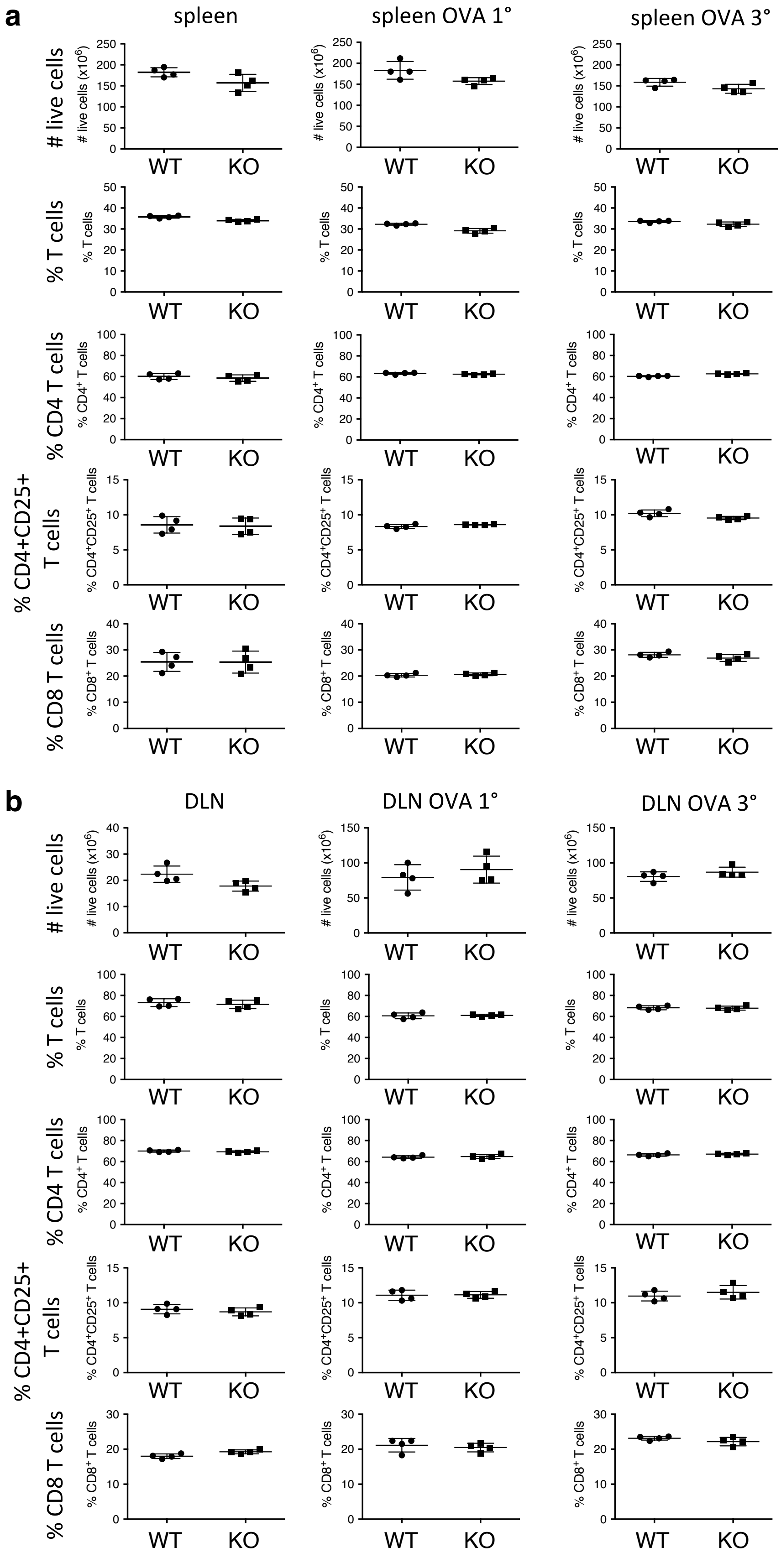

Supplementary Figure 4

Supplementary Figure 4. Immunophenotyping of rat spleen (**a**) and DLN (**b**) cells following OVA immunization. Flow cytometric analysis was used to enumerate T cell populations from WT and *Kcna3*$^{-/-}$ rats following primary immunization with OVA or after 3 rounds of OVA immunization. Individual animals (n = 4 biological replicates per group) are represented by discrete symbols and are shown with mean ± s.d. No statistically significant differences as determined by Student's *t* test were observed between WT and KO.

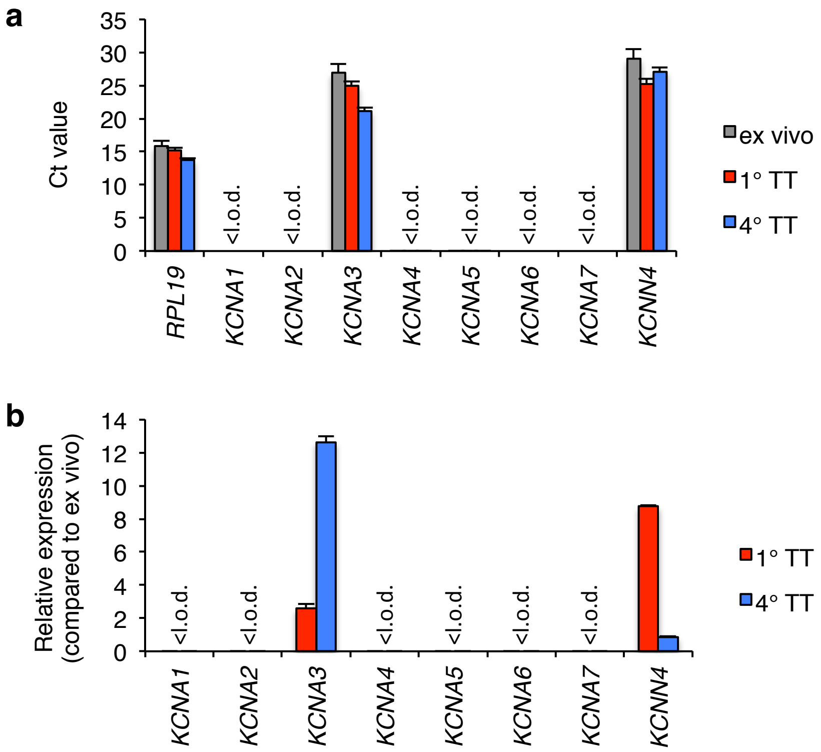

Supplementary Figure 5. K^+ channel expression in repeatedly stimulated TT-specific human T cells. Gene expression of Kv1 family members (*KCNAx*) and KCa3.1 (*KCNN4*) was measured in primary tetanus toxoid-stimulated T cells (1° TT) or T cells that underwent four rounds of TT stimulation (4° TT). (**a**) Raw Ct values, with only Kv1.3 and KCa3.1 having Ct values less than 33. (**b**) Relative expression of K^+ channels shown in comparison to *ex vivo* T cells. Data are shown as mean ± s.d. of triplicate measurements from one representative experiment. "<l.o.d." denotes below limit of detection.

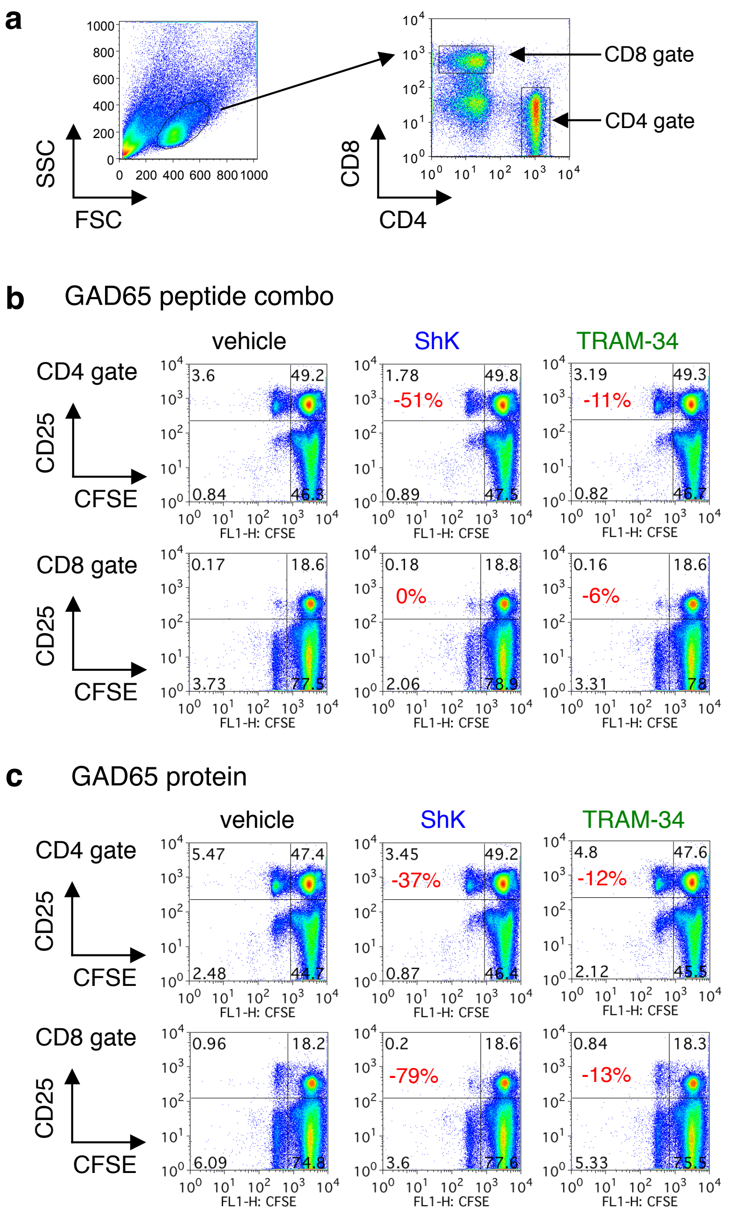

Supplementary Figure 6. ShK inhibits Type 1 diabetes autoantigen-specific CD4 and CD8 T cell proliferation. a. $CD4^+$ and $CD8^+$ T cell gating strategy. Live lymphocyte gate was set based on forward scatter (FSC) vs. side scatter (SSC). $CD4^+$ and $CD8^+$ gates were then set based on CD4 and CD8 expression, respectively. PBMC from HLA-$DR4^+$ T1D donors were stimulated with a combination of HLA-DR4-restricted GAD65 peptides (**b,** n = 10 biological replicates) in the absence or presence of either 10 nM ShK or 1 μM TRAM-34, or PBMC from non-HLA- or HLA-typed T1D donors were stimulated with GAD65 protein (**c**, n = 13 biological replicates). Proliferation responses were determined at day 7 by CFSE dilution of $CD4^+$- or $CD8^+$-gated cells. Representative data from one donor are shown. Percentages indicated in red denote % reduction in proliferation relative to vehicle-treated cells.

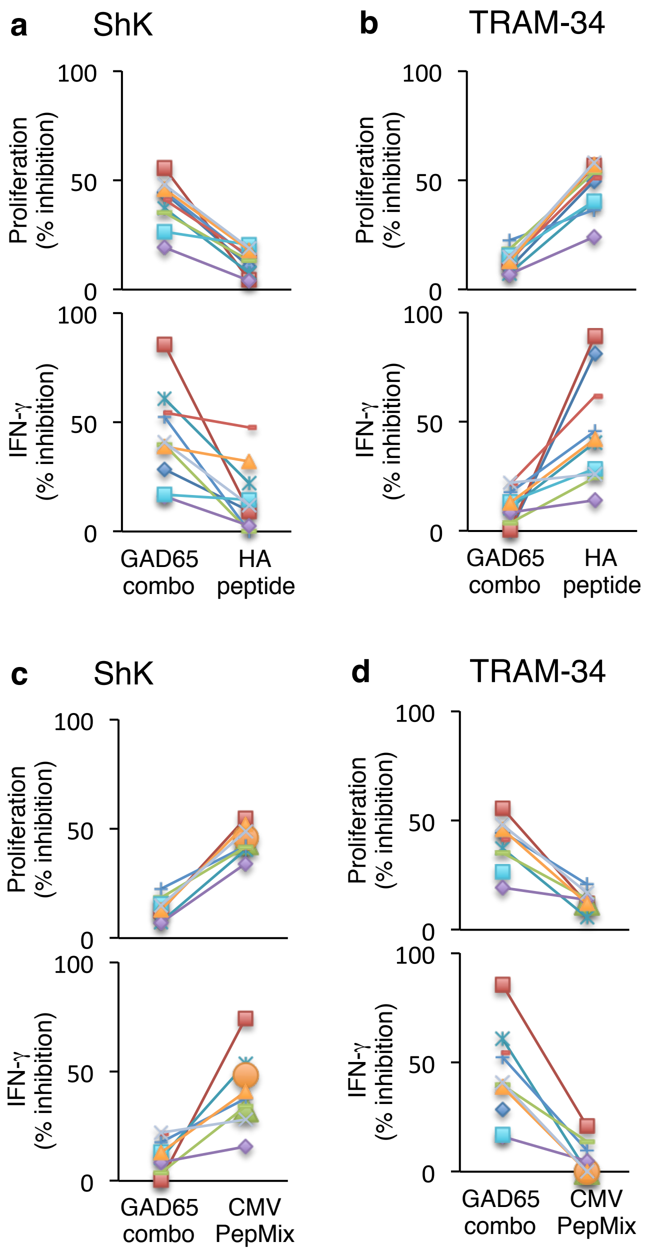

Supplementary Figure 7. ShK inhibits GAD65-specific T1D T cells but not autologous HA- or CMV-specific T cells. PBMC from T1D donors (n = 9) were stimulated with a combination of four HLA-DR4-restricted GAD65 peptides. Proliferation and IFN-γ responses were compared to HA (**a, b**) or CMV (**c, d**) stimulation in the absence or presence of either 10 nM ShK (**a, c**) or 1 μM TRAM-34 (**b, d**). Data shown are % inhibition of proliferation response as determined by ^{3}H-thymidine incorporation after 4 days primary *in vitro* stimulation and % inhibition of IFN-γ production after 3 days stimulation. Colored symbols and corresponding lines represent each individual donor.

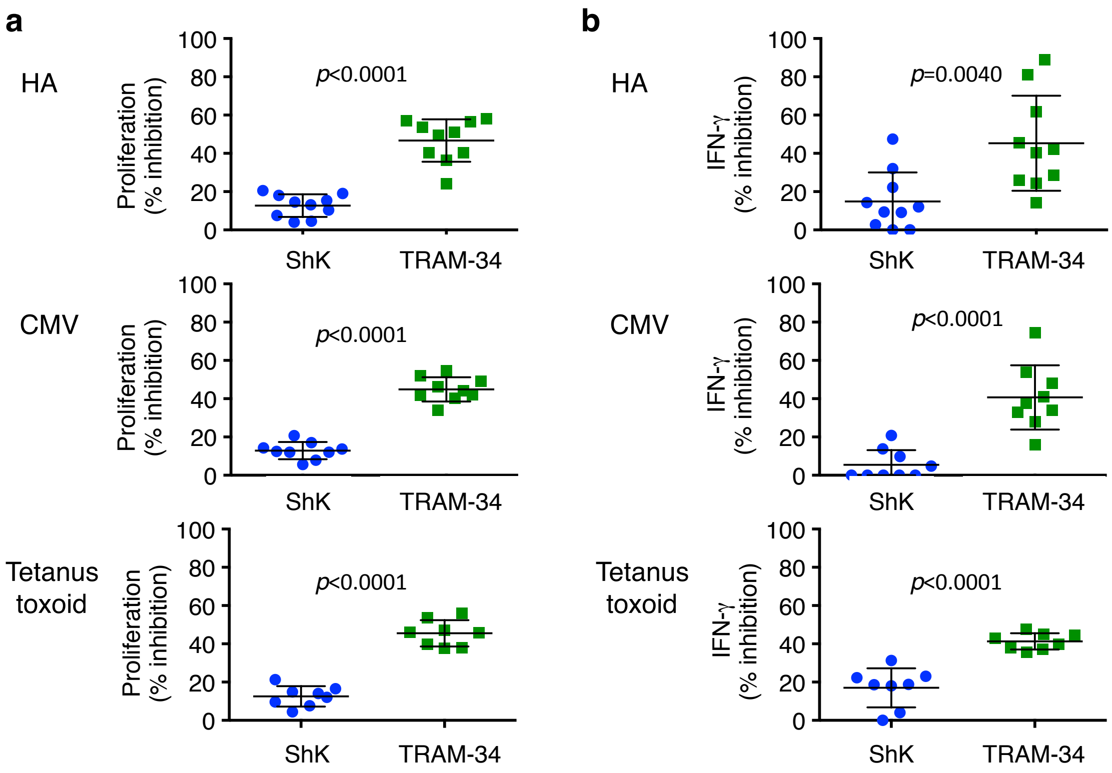

Supplementary Figure 8. T1D donor pathogen-specific T cell responses are not inhibited by ShK. PBMC from T1D donors were stimulated with HA peptide (n = 10 biological replicates), CMV PepMix (n = 9) or tetanus toxoid (n = 9) in the absence or presence of either 10 nM ShK or 1 µM TRAM-34. (**a**) Inhibition of proliferation response as determined by ^{3}H-thymidine incorporation after 4 days primary *in vitro* stimulation. (**b**) Inhibition of IFN-γ production after 3 day stimulation. Data are shown as individual data points together with mean ± s.d. Statistically significant differences are denoted with *p*-values as determined by Student's *t* test.

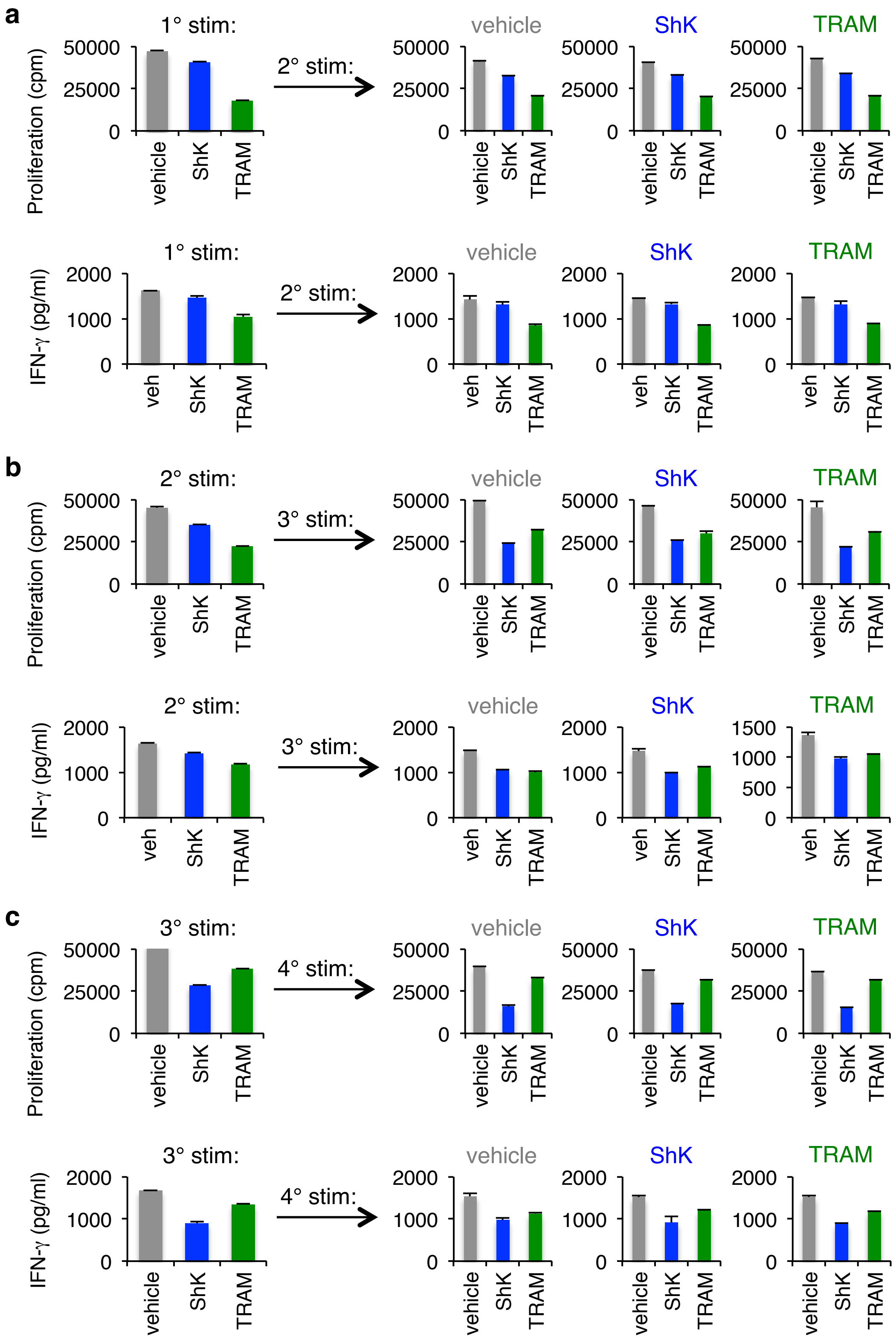

Supplementary Figure 9.

Supplementary Figure 9. Sensitivity to channel blockers is dependent on repeated stimulation and is not affected by prior exposure to inhibitor. **(a)** Primary (1°) T cell stimulation with TT was performed in the absence or presence of either 10 nM ShK or 1 μM TRAM-34. After 4 days in culture, inhibitor was washed out, cells were rested for 3 days, then restimulated a second time (2° stim) with TT and irradiated autologous PBMC in the presence of the indicated inhibitor. Proliferation responses (upper panels) were determined by addition of ^{3}H-thymidine one day prior to liquid scintillation counting which was performed on day 4. IFN-γ concentrations (lower panels) in supernatants harvested 3 days after stimulation were determined by ELISA. Data are shown as mean ± s.d. of duplicate wells. **(b)** 2° TT-specific T cells subjected to inhibitor washout and 3° TT stimulation. **(c)** 3° TT-specific T cells subjected to inhibitor washout and 4° TT stimulation.
